# Supplementary material for: Low-latency time-of-flight non-line-of-sight imaging at 5 frames per second
Source: Nat Commun. 2021 Nov 11;12:6526. doi: 10.1038/s41467-021-26721-x (PMC8586255; doi:10.1038/s41467-021-26721-x)
Supplement: Supplementary file 1 — Supplementary Information [file 41467_2021_26721_MOESM1_ESM.pdf]

Supplementary Information

Low-latency time-of-flight non-line-of-sight imaging at 5 frames per second

Ji Hyun Nam[1], Eric Brandt[2,*], Sebastian Bauer[3,*], Xiaochun Liu[1], Marco Renna[4], Alberto Tosi[4], Eftychios Sifakis[2], Andreas Velten[1,3]

[1]*Department of Electrical and Computer Engineering, University of Wisconsin Madison*
[2]*Department of Computer Science, University of Wisconsin Madison*
[3]*Department of Biostatistics and Medical Informatics, University of Wisconsin Madison*
[4]*Dipartimento di Elettronica Informazione e Bioingegneria, Politecnico di Milano*
∗*highlights equal contribution*

This supplementary document provides background information about the signal in NLOS imaging and a theoretical treatment of the resulting SNR. It also shows details from the videos and provides a list of symbols used both in the main paper and this document.

The document structure is as follows:

1 Supplementary Note 1: Comparison of diffuse and retroreflective objects 2

2 Supplementary Note 2: Intensity arriving at point object 2

3 Supplementary Note 3: NLOS imaging SNR analysis 5

3.1 NLOS reconstruction using Rayleigh Sommerfeld Diffraction (RSD) 7

3.2 Stochastic Preliminaries 9

3.3 Physical Preliminaries 11

3.4 RSD SNR analysis 13

3.4.1 Incorporating background noise 20

3.5 SNR of arbitrary linear NLOS reconstruction 20

3.6 SNR Plots 20

4 Supplementary Results 24

5 List of used symbols 27

1 Supplementary Note 1: Comparison of diffuse and retroreflective objects

We want to illustrate the much brighter signal levels created in confocal NLOS imaging of retroreflective objects compared to NLOS imaging of conventional diffuse objects. In order to compare the diffuse objects used in this paper with retroreflective ones, we set up an illustrative scene in a dark room shown in Fig. 1: it contains the two hodded sweatshirts and the stuffed toy used in the videos, as well as two retroreflective street signs and retroreflective tape. The top image is taken with room lights on and no camera flash, and the bottom image is taken with room lights off and camera flash. The latter resembles confocal NLOS acquisition, because the used cellphone camera has the flash and the camera aperture very close together, about 1.5 cm. As can be seen, the retroreflective objects send so much more light back to the camera (which mimics the relay wall) that the diffuse objects cannot be resolved by the camera and are completely dark, which shows that the objects we used indeed reflect very little light compared to retroreflective objects.

2 Supplementary Note 2: Intensity arriving at point object

This section explains that the signal characteristic of $1/r^4$ is only a worst-case scenario for distances r that are large with respect to the relay wall dimensions. For this reason, let us investigate the intensity arriving at a point object for different relay wall sizes. As in the main paper, the relay wall has the coordinates $\mathbf{x}_\mathrm{p} = (x_\mathrm{p}, y_\mathrm{p}, 0)$, and the object, without loss of generality, is located perpendicular to the origin in the relay wall plane at $\mathbf{x}_\mathrm{v} = (0, 0, z_\mathrm{v})$. For a perfectly diffuse relay wall, the light intensity reflected into a direction at an angle θ with respect to the relay wall normal is given by Lambert's cosine law [1]

$$I_\mathrm{sr} = I_\mathrm{sr0} \cos(\theta), \tag{1}$$

where the intensities I_sr, I_sr0 are given in $\mathrm{W/m^2/sr}$, i.e., I_sr0 is the intensity at the relay wall divided by 2π, so I_sr0=$I_0/2\pi$, with I_0 the usual optical intensity (in $\mathrm{W/m^2}$) at the relay wall. In NLOS imaging, the relay wall usually is scanned, which means each point is individually illuminated in sequence temporally and therefore the intensities at the object position add linearly to yield the total intensity of the object's illumination. (We want to remark that such a straightforward addition of intensities would be an oversimplification if all relay wall points would be illuminated at the same time, because the electric field contributions from different points might cancel, leading to a lower total intensity.) The total intensity at the object is therefore calculated by summing up the

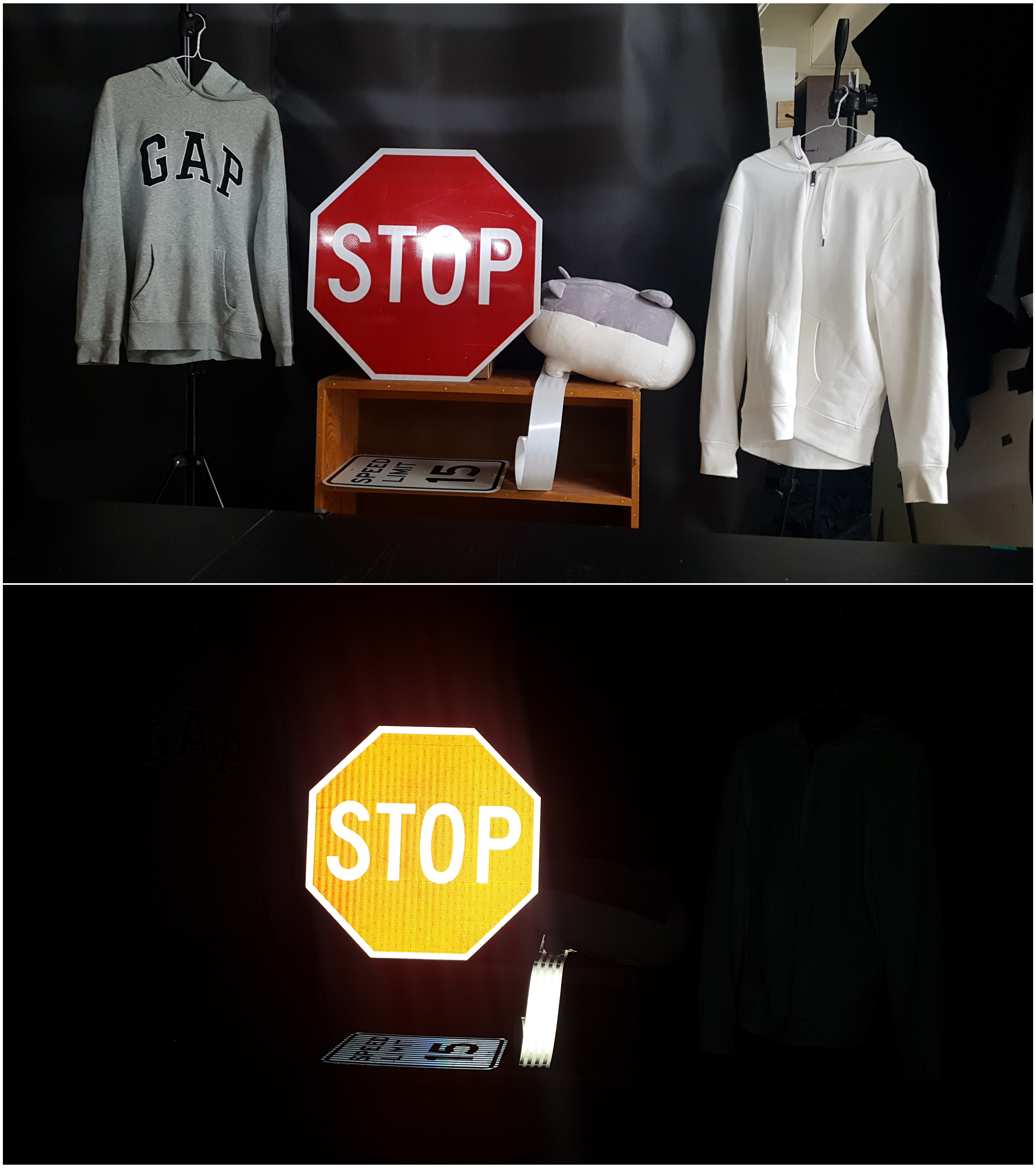

Supplementary Figure 1: Scene containing the two hooded sweatshirts and the stuffed toy used in this paper. In addition, the scene has two retroreflective street signs and retroreflective tape. The images have been taken in a dark room. Top: image with room lights on, no camera flash; bottom: room lights off, camera flash.

contributions from all relay wall positions; assuming the scanning covers the whole wall surface without gaps, this results in the integral

$$\begin{aligned} I(0,0,z_\mathrm{v}) &= \iint\limits_P \frac{I_0}{2\pi} \frac{1}{\left(\sqrt{x_\mathrm{p}^2 + y_\mathrm{p}^2 + z_\mathrm{v}^2}\right)^2} \cos(\theta)\, dx_\mathrm{p} dy_\mathrm{p} \\ &= \frac{I_0}{2\pi} \iint\limits_P \frac{1}{x_\mathrm{p}^2 + y_\mathrm{p}^2 + z_\mathrm{v}^2} \cos\left(\arctan\left(\frac{\sqrt{x_\mathrm{p}^2 + y_\mathrm{p}^2}}{z_\mathrm{v}}\right)\right) dx_\mathrm{p} dy_\mathrm{p} \\ &= \frac{I_0}{2\pi} \iint\limits_P \frac{1}{x_\mathrm{p}^2 + y_\mathrm{p}^2 + z_\mathrm{v}^2} \frac{1}{\sqrt{1 + \frac{x_\mathrm{p}^2 + y_\mathrm{p}^2}{z_\mathrm{v}^2}}} dx_\mathrm{p} dy_\mathrm{p} \\ &= \frac{I_0}{2\pi} z_\mathrm{v} \iint\limits_P \left(x_\mathrm{p}^2 + y_\mathrm{p}^2 + z_\mathrm{v}^2\right)^{-3/2} dx_\mathrm{p} dy_\mathrm{p}\,, \end{aligned} \tag{2}$$

where the identity $\cos(\arctan(x)) = 1/\sqrt{1+x^2}$ has been used and the region P denotes the relay wall area that is illuminated.

- When the relay wall is infinite, $-\infty < x_\mathrm{p}, y_\mathrm{p} < \infty$, we have

$$I(0,0,z_\mathrm{v}) = \frac{I_0}{2\pi} z_\mathrm{v} \int\limits_{-\infty}^{\infty} \int\limits_{-\infty}^{\infty} \left(x_\mathrm{p}^2 + y_\mathrm{p}^2 + z_\mathrm{v}^2\right)^{-3/2} dx_\mathrm{p} dy_\mathrm{p} = I_0\,, \tag{3}$$

  which has remarkable consequences: the intensity arriving at the point object is independent of z_v, the distance between relay wall and object. That means that in this case, there is no intensity loss between relay wall and object, which is physically sound because the total energy propagating into the half space away from the relay wall must not change. Due to the fact that the object sends back a spherical wave to the relay wall, there is a total intensity falloff of only $1/r^2$.

- For an infinitesimally small relay wall, i.e., just one point, the integration that needs to be performed is given by

$$I(0,0,z_\mathrm{v}) = \frac{I_0}{2\pi} z_\mathrm{v} \int\limits_{-\infty}^{\infty} \int\limits_{-\infty}^{\infty} \left(x_\mathrm{p}^2 + y_\mathrm{p}^2 + z_\mathrm{v}^2\right)^{-3/2} \delta(x_\mathrm{p}, y_\mathrm{p}) dx_\mathrm{p} dy_\mathrm{p} = \frac{I_0}{2\pi} z_\mathrm{v} \left(z_\mathrm{v}^2\right)^{-3/2} = \frac{I_0}{2\pi z_\mathrm{v}^2}\,, \tag{4}$$

  and incorporating the loss along the return path results in the total intensity falloff of $1/r^4$.

- For the most relevant practical case of a rectangular relay wall, the falloff from the relay wall to the object will be in between the extreme cases of a constant and $1/r^2$:

$$I(0,0,z_\mathrm{v}) = \frac{I_0}{2\pi} z_\mathrm{v} \int_{-b}^{b} \int_{-a}^{a} \left(x_\mathrm{p}^2 + y_\mathrm{p}^2 + z_\mathrm{v}^2\right)^{-3/2} dx_\mathrm{p} dy_\mathrm{p} = \frac{2I_0}{\pi} \arctan\left(\frac{ab}{z_\mathrm{v}\sqrt{a^2+b^2+z_\mathrm{v}^2}}\right). \tag{5}$$

  For a square relay wall, $b = a$, this can be rewritten

$$I(0,0,z_\mathrm{v}) = \frac{I_0}{2\pi} \arctan\left(\frac{a^2}{z_\mathrm{v}\sqrt{2a^2+z_\mathrm{v}^2}}\right) = \frac{2I_0}{\pi} \arctan\left(\frac{\frac{a^2}{z_\mathrm{v}^2}}{\sqrt{2\frac{a^2}{z_\mathrm{v}^2}+1}}\right). \tag{6}$$

  For large $z_\mathrm{v} \gg a$, the $\arctan$ argument is very small and the Taylor series approximation $\arctan(x) \approx x$ can be used, again leading to a characteristic proportional to $1/r^2$.

Supplementary Figure 2 shows the intensity arriving at the point object as a function of the object distance z_v and relay wall side length a. From (6), we see that only the relative ratio between relay wall size a and object distance z_v is important, not the absolute numbers, and therefore the bottom plot of Supplementary Fig. 2 gives the fraction of the total relay wall illumination intensity that arrives at the point object.

3 Supplementary Note 3: NLOS imaging SNR analysis

The SNR analysis of NLOS imaging is crucial in determining its capabilities and limits. Existing publications on NLOS imaging[2,3] only consider the fall-off of the signal with increasing distance between object and relay wall, which roughly follows a $1/r^4$ curve for large r which is approximately the perpendicular distance between the relay wall and the object. However, in order to adequately assess the image quality of NLOS imaging systems, it is necessary to also analyze the noise contribution. We will derive the SNR for NLOS imaging below. Here, we again point out the analogy between conventional photography and seeing around corners[4]: a conventional camera pixel images (i.e., it integrates light from) an area that increases with r^2, which means that at larger distances, it has lower resolution, see Supplementary Figure 3 and similarly Figure 4e in the main paper. NLOS image reconstruction is conventionally performed on a Cartesian reconstruction grid having a constant lateral resolution at all distances. Similar to conventional imaging, however, like any NLOS reconstruction method, the RSD approach we use has a Point Spread Function (PSF) that becomes wider with increasing distance. We utilize this fact by applying depth-dependent averaging: the further away a reconstruction plane, the more corresponding planes from neighboring frames are averaged. This has a beneficial effect on both moving and stationary objects: the SNR

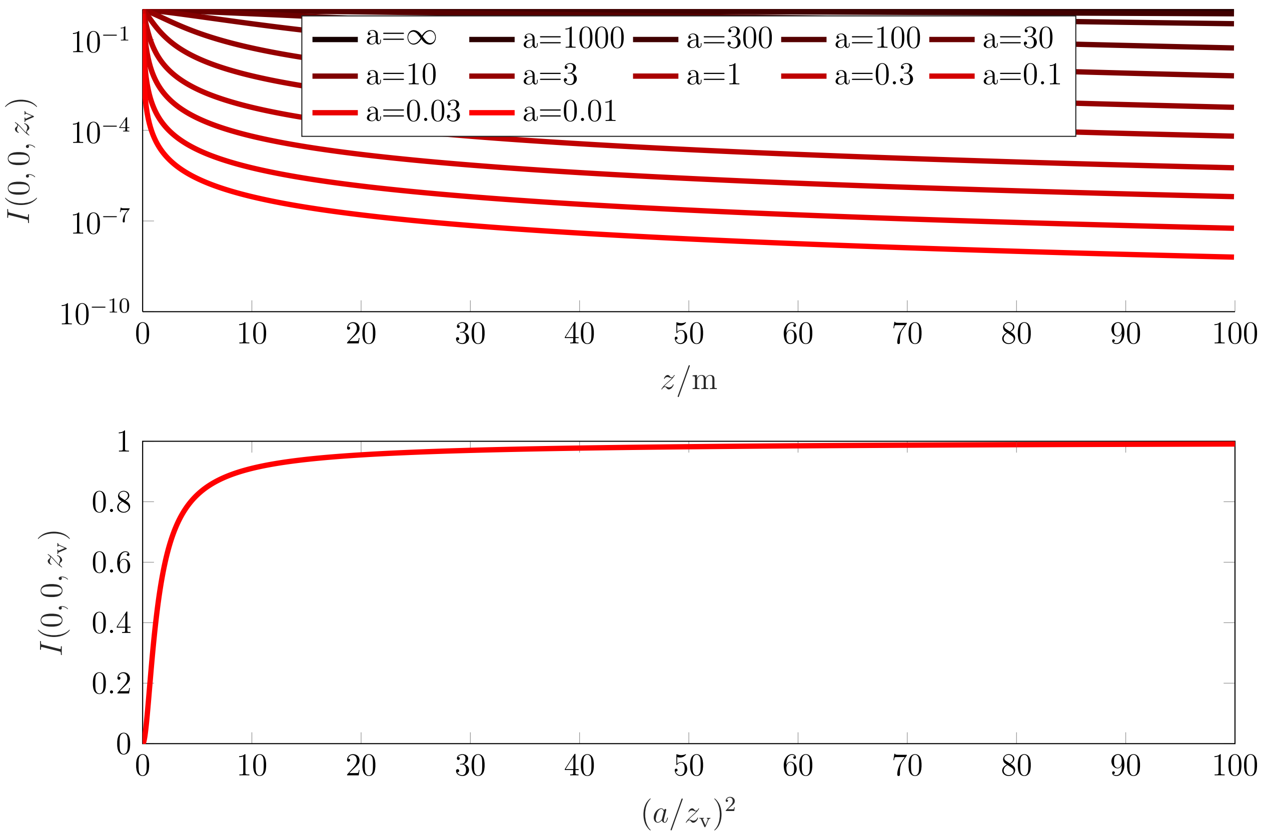

Supplementary Figure 2: Top: Intensity arriving at a point object located on the optical axis at distance z_v from the relay wall for $I_0 = 1$. For a large relay wall, $a \to \infty$, there is no intensity loss; when the relay wall is merely a point, the intensity falls proportionally to $\frac{1}{z_v^2}$. Bottom: This time, the intensity is plotted against the ratio between the relay wall side length a and the distance z_v (cf. (6)). For example, when a is ten times larger than the object distance, about 91 % of the total intensity arrive at the object.

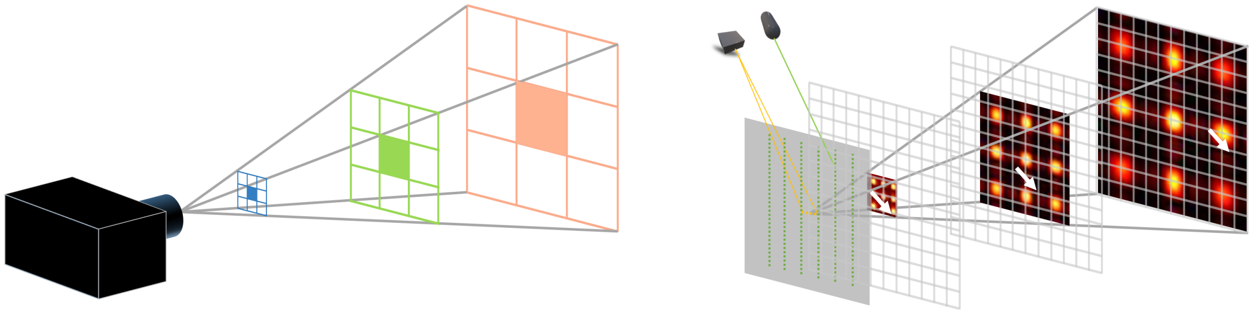

Supplementary Figure 3: Depth-dependent area covered by the pixels of a conventional camera cover (left), actual RSD point spread function at various positions (right). While calculated on a rectangular grid, the uncertainty of NLOS reconstruction, analogously to conventional imaging, grows with r^2. The white arrows depicts the movement of 3 point objects with the same speed between two video frames. At the closest distance, the object position is blurred linearly along the arrow because of the object's motion. The point object at the farthest distance cannot be resolved within a single frame because of the spatial blur which is larger anyway, so when incorporating the time axis, more frames can be averaged at larger distances without loss of resolution. Static objects benefit from the averaging anyway. Note that depth-dependent averaging of video frames is not possible with conventional imaging where depth information is unavailable.

of stationary objects improves naturally, and objects moving less than the width of the PSF would be blurred anyway because of the reduced spatial resolution, so the averaging also improves the SNR in this case. We want to emphasize again that a loss in resolution at increasing distance is intrinsic to conventional imaging and not a downside specific to NLOS imaging.

The most relevant noise sources of a SPAD-based NLOS imaging system are natural (Poisson noise and ambient light) and arise from the hardware (dark count rate and afterpulsing). The ambient light can be greatly reduced by a suitable bandpass filter, while the rest is assumed to follow a Poisson distribution, which also includes the dark count rate which for our system is almost negligible. Afterpulsing can be reduced with sufficiently long dead-time after each avalanche detection (200 ns in our system). The dominant source of noise is the stochastic nature of photon arrival times, i.e., Poisson-distributed noise.

3.1 NLOS reconstruction using Rayleigh Sommerfeld Diffraction (RSD)

The RSD NLOS reconstruction algorithm [5] is based on interpreting the time histograms acquired by SPAD sensors as impulse response functions. Therefore, considering the fact that the light travels much faster than scene objects can move, the scene can be considered a linear and time invariant system and the response of the scene to any input signal can be calculated by convolving the input function with the measured impulse response. The chosen input function is a phasor field

pulse, a sinusoidal intensity modulation (having a typical wavelength between 4–8 centimeters) multiplied by a Gaussian to only propagate a pulse comprising few modulation periods. This input signal is considered to emerge from the stationary SPAD observation position on the relay wall (note that because of scene reciprocity, the laser and SPAD positions can be swapped). After convolving the time responses at all scanned laser relay wall positions with this input signal, we now know the response of the system to the modulated pulse. The scene reconstruction is then calculated by propagating this response wavefront back into the scene, and at the correct object locations, this wavefront will take on the shape of the object.

Using conventional notation [5], this process can be described mathematically as follows. The relay wall is illuminated at a set of points $\mathbf{x}_\mathrm{p} = (x_\mathrm{p}, y_\mathrm{p}, 0)$ (projection points) and the light returning to points $\mathbf{x}_\mathrm{c} = (x_\mathrm{c}, y_\mathrm{c}, 0)$ (camera points) is collected. The resulting impulse response is denoted $H(\mathbf{x}_\mathrm{p} \to \mathbf{x}_\mathrm{c}, t)$. For this and all other quantities, frequency domain representations are denoted by the same variable as the respective time domain quantities, but with the subscript $\mathcal{F}$ and the argument of angular frequency Ω instead of t, so the Fourier transformed version of $H(\mathbf{x}_\mathrm{p} \to \mathbf{x}_\mathrm{c}, t)$ with respect to time is $H_\mathcal{F}(\mathbf{x}_\mathrm{p} \to \mathbf{x}_\mathrm{c}, \Omega)$. The frequency domain phasor field illumination pulse is denoted $\mathcal{P}_\mathcal{F}(\mathbf{x}_\mathrm{p}, \Omega)$,

$$\mathcal{P}_\mathcal{F}(\mathbf{x}_\mathrm{p}, \Omega) = \delta(\mathbf{x}_\mathrm{p} - \mathbf{x}_\mathrm{ls}) \left(2\pi\delta(\Omega - \Omega_\mathrm{C}) \underset{f}{*} \sigma\sqrt{2\pi} e^{-\frac{\sigma^2\Omega^2}{2}} \right) = \delta(\mathbf{x}_\mathrm{p} - \mathbf{x}_\mathrm{ls}) (2\pi)^{3/2} \sigma e^{-\frac{\sigma^2(\Omega-\Omega_\mathrm{C})^2}{2}} \quad (7)$$

and the received signal at $\mathbf{x}_\mathrm{c}$ is given by $\mathcal{P}_\mathcal{F}(\mathbf{x}_\mathrm{c}, \Omega) = \mathcal{P}_\mathcal{F}(\mathbf{x}_\mathrm{p}, \Omega) H_\mathcal{F}(\mathbf{x}_\mathrm{p} \to \mathbf{x}_\mathrm{c}, \Omega)$. In (7), Ω_C is the frequency of the intensity modulation sine function and σ the inverse width of the Gaussian pulse in the time domain. The frequency domain representation $\mathcal{P}_\mathcal{F}(\mathbf{x}_\mathrm{p}, \Omega)$ of the received phasor field pulse is convenient because the Rayleigh-Sommerfeld diffraction integral only processes a single frequency component; at a reconstruction plane parallel to the relay wall, the resulting wavefront is just a 2D spatial convolution of the respective convolution kernel with the wavefront in the relay wall plane. After processing each frequency component separately, inverse Fourier transform calculates the time-dependent wavefront. The overall scene reconstruction at the voxels

$\mathbf{x}_\mathrm{v} = (x_\mathrm{v}, y_\mathrm{v}, z_\mathrm{v})$ located on the plane at depth z_v is calculated by (see [5] for details)

$$\begin{aligned}
\mathcal{I}(\mathbf{x}_\mathrm{v}, t) &= \Phi(\mathcal{P}(\mathbf{x}_\mathrm{c}, t)) \\
&= |\mathcal{P}(x_\mathrm{v}, y_\mathrm{v}, z_\mathrm{v}, t)|^2 \\
&= \left| \int_{\Omega_\mathrm{C}-\Delta\Omega}^{\Omega_\mathrm{C}+\Delta\Omega} e^{j\Omega t} \iint_{-\infty}^{+\infty} \mathcal{P}_\mathcal{F}(x_\mathrm{c}, y_\mathrm{c}, 0, \Omega) \underbrace{\frac{e^{-j\frac{\Omega}{c}\sqrt{(x_\mathrm{c}-x_\mathrm{v})^2+(y_\mathrm{c}-y_\mathrm{v})^2+z_\mathrm{v}^2}}}{\sqrt{(x_\mathrm{c}-x_\mathrm{v})^2+(y_\mathrm{c}-y_\mathrm{v})^2+z_\mathrm{v}^2}}}_{\text{RSD diffraction kernel}} \, dx_\mathrm{c}\, dy_\mathrm{c} \frac{d\Omega}{2\pi} \right|^2 \\
&= \left| \int_{\Omega_\mathrm{C}-\Delta\Omega}^{\Omega_\mathrm{C}+\Delta\Omega} e^{j\Omega t} \iint_{-\infty}^{+\infty} \mathcal{P}_\mathcal{F}(x_\mathrm{c}, y_\mathrm{c}, 0, \Omega) \cdot \underbrace{G(x_\mathrm{v}-x_\mathrm{c}, y_\mathrm{v}-y_\mathrm{c}, z_\mathrm{v}, \Omega)}_{\text{2D convolution kernel}} \, dx_\mathrm{c}\, dy_\mathrm{c} \frac{d\Omega}{2\pi} \right|^2 \\
&= \left| \int_{\Omega_\mathrm{C}-\Delta\Omega}^{\Omega_\mathrm{C}+\Delta\Omega} e^{j\Omega t} \underbrace{\mathcal{P}_\mathcal{F}(x_\mathrm{c}, y_\mathrm{c}, 0, \Omega) * G(x_\mathrm{c}, y_\mathrm{c}, z_\mathrm{v}, \Omega)}_{\text{Spatial 2D convolution}} \frac{d\Omega}{2\pi} \right|^2 .
\end{aligned} \tag{8}$$

In the textbook RSD, $G(x_\mathrm{c}, y_\mathrm{c}, z_\mathrm{v}, \Omega) = \frac{\alpha(x_\mathrm{v}, y_\mathrm{v}, z_\mathrm{v}) \cdot \exp(-j\frac{\Omega}{c}\sqrt{x_\mathrm{c}^2+y_\mathrm{c}^2+z_\mathrm{v}^2})}{\sqrt{x_\mathrm{c}^2+y_\mathrm{c}^2+z_\mathrm{v}^2}}$ is the 2D convolution kernel for electromagnetic wave propagation, but the factor $\alpha(x_\mathrm{v}, y_\mathrm{v}, z_\mathrm{v})$ will be ignored during NLOS reconstruction as it only affects the reconstruction brightness, but not the location of the reconstructed objects. Furthermore, only the angular frequencies in the interval $\Omega \in [\Omega_\mathrm{C} - \Delta\Omega, \Omega_\mathrm{C} + \Delta\Omega]$ are considered; the very small magnitudes outside this interval are neglected. To further reduce the computation time, the time variable t is replaced by a certain formula of spatial variables[5]. We want to remark that the convolution integral over the finite relay wall aperture $dx_\mathrm{c}\, dy_\mathrm{c}$ obviously should not be calculated from $-\infty$ to ∞, in the continuous (integral) case; this is just done here for illustration of the convolution. When numerically calculating the convolution on a computer using the 2D FFT (Fast Fourier Transform), multiplication and inverse 2D FFT, this is fine, as the FFT implicitly assumes periodic extension.

3.2 Stochastic Preliminaries

The analysis of the SNR in NLOS imaging has to start at the beginning; the light source. The number of photons emitted by the laser in each pulse (more precisely, within all disjoint time intervals during one pulse) is a random number following a Poisson distribution. This means that everything that happens later, i.e., reflection off the relay wall, the objects, and the relay wall again, also has to be treated in a stochastic way and all calculations have to be performed with random variables. The following considerations are based on the SNR defined by

$$\mathrm{SNR} = \frac{\mu}{\sigma}, \tag{9}$$

where μ denotes the mean of a random variable and σ its standard deviation. In our case, we are interested in the SNR at each reconstruction voxel, which means we have to calculate the mean and the variance of the reconstruction value (brightness) of a specific voxel. This can be performed as a function of the mean and the variance of the laser pulse. It is of course possible to describe the forward model (integration along spheroids) and the RSD reconstruction from Eq. (8) as a sequence of integrals and to calculate mean and variance at a specific reconstruction position from them. However, this is very cumbersome even for just a point object and there might not be an analytical solution, as from one integral to the next, the integrands become more complicated. Instead, we choose a discrete model which is based on simulated scenes; repeated simulations of the same scene with different noise realizations in each run are added to the ground truth signal, and from all reconstructions sample mean and variance are calculated to determine the SNR (9). The presented model allows the use of all reconstruction methods that can be represented as a matrix multiplication (for example the RSD, backprojection and others).

Let us briefly introduce some tools for the stochastic description of NLOS imaging. Let $\mathbf{X} \in \mathbb{R}^K$ be a real random vector that is multiplied by a constant complex matrix $\mathbf{A} \in \mathbb{C}^{M \times K}$, $\mathbf{Y} = \mathbf{AX}$. The resulting mean vector is calculated from

$$\mathbb{E}\{\mathbf{Y}\} = \mathbb{E}\{\mathbf{AX}\} = \Re(\mathbb{E}\{\mathbf{AX}\}) + j\Im(\mathbb{E}\{\mathbf{AX}\}) = \Re(\mathbf{A})\,\mathbb{E}\{\mathbf{X}\} + j\Im(\mathbf{A})\,\mathbb{E}\{\mathbf{X}\}\,. \tag{10}$$

The covariance matrix of $\mathbf{X}$ in general is calculated by

$$\mathbf{K_{XX}} = \mathbb{E}\{(\mathbf{X} - \mathbb{E}\{\mathbf{X}\})(\mathbf{X} - \mathbb{E}\{\mathbf{X}\})^{\mathrm{H}}\} = \mathbb{E}\{(\mathbf{X} - \overline{\mathbf{X}})(\mathbf{X} - \overline{\mathbf{X}})^{\mathrm{H}}\}\,, \tag{11}$$

where $(\cdot)^{\mathrm{H}}$ denotes the conjugate transpose and we introduce $\overline{\mathbf{X}} = \mathbb{E}\{\mathbf{X}\}$. The covariance matrix $\mathbf{K_{YY}}$ of $\mathbf{Y}$ can then be expressed by

$$\begin{aligned}
\mathbf{K_{YY}} &= \mathbb{E}\{(\mathbf{Y} - \overline{\mathbf{Y}})(\mathbf{Y} - \overline{\mathbf{Y}})^{\mathrm{H}}\} \\
&= \mathbb{E}\{(\mathbf{AX} - \mathbf{A}\overline{\mathbf{X}})(\mathbf{AX} - \mathbf{A}\overline{\mathbf{X}})^{\mathrm{H}}\} \\
&= \mathbb{E}\{(\mathbf{A}(\mathbf{X} - \overline{\mathbf{X}}))(\mathbf{A}(\mathbf{X} - \overline{\mathbf{X}}))^{\mathrm{H}}\} \\
&= \mathbb{E}\{(\mathbf{A}(\mathbf{X} - \overline{\mathbf{X}}))((\mathbf{X} - \overline{\mathbf{X}})^{\mathrm{H}}\mathbf{A}^{\mathrm{H}})\} \\
&= \mathbf{A}\,\mathbb{E}\{((\mathbf{X} - \overline{\mathbf{X}}))((\mathbf{X} - \overline{\mathbf{X}})^{\mathrm{H}})\}\mathbf{A}^{\mathrm{H}} \\
&= \mathbf{A}\mathbf{K_{XX}}\mathbf{A}^{\mathrm{H}}\,.
\end{aligned} \tag{12}$$

For calculating higher-order expected values, moment-generating functions are a convenient tool. The moment-generating function of a random variable X is defined by

$$M_X(s) = \mathbb{E}\{e^{sX}\}\,, \tag{13}$$

and for the linear combination $S_K = \sum_{k=1}^{K} a_k X_k$ of independent random variables X_k, the moment-generating function is the product of the moment-generating functions with scaled arguments:

$$M_{S_K}(s) = M_{X_1}(a_1 s) M_{X_2}(a_2 s) \cdots M_{X_K}(a_K s)\,. \tag{14}$$

The expected values are then calculated by

$$\mathbb{E}\{X^k\} = \left. \frac{d^k M_X(s)}{ds^k} \right|_{s=0} . \tag{15}$$

3.3 Physical Preliminaries

The light emitted by a laser follows a Poisson distribution. To not further complicate things, we assume that the laser pulse is shorter than a SPAD time bin width. If that is not the case, the photon rate can be described by an inhomogeneous Poisson point process, where the photon rate within disjoint time intervals is in general varies, but still follows a Poisson distribution. In the considered scenario, we can therefore assume the random variable $\mathrm{Ph}_{\text{laser}}$ describing the number of photons emitted by the laser in a time period Δt (SPAD bin width) during the pulse follows the Poisson probability mass function given by

$$P\left\{\mathrm{Ph}_{\text{laser}} = n\right\} = \frac{\Lambda^n}{n!} e^{-\Lambda} , \tag{16}$$

where $\Lambda = \lambda \Delta t$ (the expected value of photons during time interval Δt) and λ is the expected rate, i.e., the number of photons per time interval.

Let us analyze in more detail how this distribution arises because it is very important for all subsequent derivations. The Poisson distribution is actually a special case of the binomial distribution, so let us explain this one first. The binomial distribution analyzes a sequence of K *independent* experiments with a binary outcome. In our case, the outcome is either that there is a photon arriving in a very short time interval δt or there is no photon arriving in this interval; δt is chosen so small that no more than one photon can arrive. We can divide the time interval Δt into a very large number K of time intervals of length δt, which means we will on average detect Λ/K photons during an interval of length δt. The binomial distribution is the discrete probability distribution of registering a certain number of photons during the full interval Δt. Taking the limit as K goes to infinity makes one arrive at the Poisson distribution.

It is very important to stress that these single photon events are *independent* of each other, which means that they have no influence on each other. What happens if the laser beam hits a surface and we are interested in the number of photons scattering in a certain direction? On average, we know that the fraction α of incident photons is reflected into this direction. Going back to our consideration of small time intervals δt, it is now less likely that we will receive a photon during one interval. We don't know which photons exactly will be eliminated from the photon stream because they are either absorbed or scattered into another direction, but we know that the average number of photons during Δt will reduce by α, so Λ will reduce to $\alpha\Lambda$. Because of the independence of the photon events, they don't influence each other (there might be other effects during scattering which we neglect; there is a large amount of publications studying scattering

in detail for certain cases, and we want to have a generic high-level description suitable for this application), so the other photons don't care if a photon is removed from the stream or not. As another consequence, the measurements in each time bin are independent of all other bins. The only constraint that we have to consider is that the total number of photons emitted by the laser has to equal the total number of photons reflected in all directions plus the number of absorbed photons. Because of the fact that most photons are not absorbed, this latter constraint can be set to zero.

It is important to note that the parameter Λ of the Poisson distribution (16) at the same time is its mean value and its variance, so $\mu_{\text{Poisson}} = \sigma^2_{\text{Poisson}} = \Lambda$. According to Eq. (9), the SNR of a Poisson-distributed random variable is given by

$$\text{SNR} = \frac{\mu}{\sigma} = \frac{\Lambda}{\sqrt{\Lambda}} = \sqrt{\Lambda}\,, \tag{17}$$

so it increases with the square root of the expected number of photons.

Let us now introduce the rest of the notation. As before, the laser illumination position on the relay wall ("projector") is denoted by the vector $\mathbf{x}_\text{p}$ having the elements $\mathbf{x}_\text{p} = (x_\text{p}, y_\text{p}, 0)$ and the SPAD ("camera") observation point is given by $\mathbf{x}_\text{c} = (x_\text{c}, y_\text{c}, 0)$. The reconstruction voxels ("volume") have the coordinates $\mathbf{x}_\text{v} = (x_\text{v}, y_\text{v}, z_\text{v})$. The distance d_1 is the distance from the physical laser to the laser position on the relay wall, d_2 is the distance from the laser position on the relay wall to the point object, d_3 the distance from the object to the SPAD observation point on the relay wall and d_4 the distance from the relay wall SPAD detection position to the position of the physical SPAD detector.

For the following, we assume that the relay wall reflects the light with an attenuation factor α in all directions, and the target reflects light with an attenuation factor β, also isotropically. These values could be adapted to being spatially dependent and having directivity, but that does not change the principle described in the following. In our case, the laser emits multiple pulses and the SPAD counts at most one photon per pulse, but let us assume all those processes happen during one pulse (the sum of Poisson-distributed random variables is a random variable that also follows a Poisson distribution with the total parameter Λ_{total} equaling the sum of the parameters of the sum elements). The pulse hits the relay wall, where only a fraction α of the photons is reflected and scatters in all directions. At the object, only the fraction $\frac{\alpha}{2\pi d_2^2}$ of the original number of photons arrives on average (we want to remark that the size of the reflective object surface was omitted; $\frac{1}{2\pi d^2}$ only provides the intensity per area, not intensity itself because that would require the multiplication by the object area). Note that the SNR decreases, as the SNR of the photon stream at the object before reflection calculates to

$$\text{SNR}_{\text{object}} = \frac{\mu_{\text{object}}}{\sigma_{\text{object}}} = \frac{\frac{\alpha}{2\pi d_2^2}\Lambda}{\sqrt{\frac{\alpha}{2\pi d_2^2}\Lambda}} = \sqrt{\frac{\alpha}{2\pi d_2^2}\Lambda}\,. \tag{18}$$

Repeating this process for the reflection off the object and for the second reflection off the relay wall yields the following parameter for the Poisson-distributed number of photons arriving at the detector within the respective time interval:

$$\Lambda_{\text{det}} = \frac{\alpha^2 \beta}{8\pi^3 d_2^2 d_3^2 d_4^2} \Lambda = \mu_{\text{det}} = \sigma_{\text{det}}^2 \,. \tag{19}$$

Let us define the attenuation factor $\gamma(\mathbf{x}_\text{p}, \mathbf{x}_\text{c}, \mathbf{x}_\text{v})$

$$\gamma(\mathbf{x}_\text{p}, \mathbf{x}_\text{c}, \mathbf{x}_\text{v}) = \begin{cases} \frac{\alpha^2 \beta}{64\pi^3 \|\mathbf{x}_\text{p} - \mathbf{x}_\text{v}\|^2 \|\mathbf{x}_\text{c} - \mathbf{x}_\text{v}\|^2 d_4^2}, & \text{when there is an object at voxel } \mathbf{x}_\text{v}, \\ 0, & \text{otherwise}\,. \end{cases} \tag{20}$$

Note that d_4 is constant and therefore not an argument of $\gamma(\mathbf{x}_\text{p}, \mathbf{x}_\text{c}, \mathbf{x}_\text{v})$. Strictly speaking, the Poisson probability mass function is not defined for $\Lambda = 0$, but in the following, we will set the value of a random variable with such parameter to zero.

The SPAD detects the travel time of each photon and builds a histogram. The arriving photons are placed in the histogram bin corresponding to the time delay according to the total distance the photons traveled (i.e., from the laser to relay wall, from relay wall to object and back to the relay wall and finally to the detector). Considering a general scene, not just a point object, the histograms are given by $H(\mathbf{x}_\text{p} \to \mathbf{x}_\text{c}, t)$, where $\mathbf{x}_\text{p}$ denotes the laser projection position and $\mathbf{x}_\text{c}$ the SPAD detection position on the relay wall. Each histogram bin at time index t contains the sum of random variables

$$H(\mathbf{x}_\text{p} \to \mathbf{x}_\text{c}, t) = \sum_{\mathbf{x}_\text{v} \in W(t)} \text{Ph}_\text{SPAD}(\mathbf{x}_\text{p}, \mathbf{x}_\text{c}, \mathbf{x}_\text{v})\,, \tag{21}$$

where $W(t)$ is the set of all points $\mathbf{x}_\text{v}$ that lie on a spheroid with foci $\mathbf{x}_\text{p}$ and $\mathbf{x}_\text{c}$ such that the travel time from $\mathbf{x}_\text{p}$ to $\mathbf{x}_\text{v}$ to $\mathbf{x}_\text{c}$ equals t. The random variables $\text{Ph}_\text{SPAD}(\mathbf{x}_\text{p}, \mathbf{x}_\text{c}, \mathbf{x}_\text{v})$ stand for the photons returning from the locations $\mathbf{x}_\text{v}$ in the 3D reconstruction space and follow a Poisson distribution with parameter $\Lambda(\mathbf{x}_\text{p}, \mathbf{x}_\text{c}, \mathbf{x}_\text{v}) = \gamma(\mathbf{x}_\text{p}, \mathbf{x}_\text{c}, \mathbf{x}_\text{v})\Lambda$. This means the histogram entry $H(\mathbf{x}_\text{p} \to \mathbf{x}_\text{c}, t)$ at time t is a Poisson-distributed random variable with parameter

$$\Lambda(\mathbf{x}_\text{p} \to \mathbf{x}_\text{c}, t) = \Lambda \sum_{\mathbf{x}_\text{v} \in W(t)} \gamma(\mathbf{x}_\text{p}, \mathbf{x}_\text{c}, \mathbf{x}_\text{v})\,. \tag{22}$$

3.4 RSD SNR analysis

We continue with common notation[4], but now let the arguments be discrete instead of continuous. This means that the time responses are indexed by t, and the total number of considered time bins is T. Ω is the frequency index.

The phasor field of frequency index Ω at position $(x_c, y_c, 0)$ that needs to be backpropagated is given by

$$\mathcal{P}_{\mathcal{F}}(x_{\mathrm{c}}, y_{\mathrm{c}}, 0, \Omega) = \mathcal{P}_{\mathcal{F}}(x_{\mathrm{p}}, y_{\mathrm{p}}, 0, \Omega) \cdot \left(\sum_{t=0}^{T-1} H(\mathbf{x}_{\mathrm{p}} \to \mathbf{x}_{\mathrm{c}}, t) e^{-j 2\pi \frac{t}{T} \Omega} \right) , \tag{23}$$

where $\mathcal{P}_{\mathcal{F}}(x_{\mathrm{p}}, y_{\mathrm{p}}, 0, \Omega)$ is the illuminating virtual phasor field pulse. Subsequently, the phasor field of frequency index Ω in the reconstruction plane at distance z_{v} is calculated by the spatial 2D convolution

$$\mathcal{P}_{\mathcal{F}}(x_{\mathrm{v}}, y_{\mathrm{v}}, z_{\mathrm{v}}, \Omega) = \sum_{x_c=-\infty}^{\infty} \sum_{y_c=-\infty}^{\infty} \mathcal{P}_{\mathcal{F}}(x_{\mathrm{c}}, y_{\mathrm{c}}, 0, \Omega) \cdot G\left(x_{\mathrm{v}} - x_{\mathrm{c}}, y_{\mathrm{v}} - y_{\mathrm{c}}, z_v, \Omega\right) . \tag{24}$$

The reconstruction at the plane with depth z_{v} at the right point in time follows from

$$\mathcal{I}(\mathbf{x}_v) = \left| \frac{1}{T} \sum_{\Omega=0}^{T} \mathcal{P}_{\mathcal{F}}(x_{\mathrm{v}}, y_{\mathrm{v}}, z_{\mathrm{v}}, \Omega) e^{j 2\pi \frac{t'}{T} \Omega} \right|^2 . \tag{25}$$

t' denotes the time index of interest, i.e., when the illumination phasor field pulse passes through the considered reconstruction voxel, with its calculation described in[5]. Inserting all this into one equation yields

$$\mathcal{I}(\mathbf{x}_v) = \left| \frac{1}{T} \sum_{\Omega=0}^{T} \left(\sum_{x_c=-\infty}^{\infty} \sum_{y_c=-\infty}^{\infty} \left(\mathcal{P}_{\mathcal{F}}(x_{\mathrm{p}}, y_{\mathrm{p}}, 0, \Omega) \cdot \left(\sum_{t=0}^{T-1} H(\mathbf{x}_{\mathrm{p}} \to \mathbf{x}_{\mathrm{c}}, t) e^{-j 2\pi \frac{t}{T} \Omega} \right) \right) \cdot G\left(x_{\mathrm{v}} - x_{\mathrm{c}}, y_{\mathrm{v}} - y_{\mathrm{c}}, z_v, \Omega\right) \right) e^{j 2\pi \frac{z_v}{Z_v} \Omega} \right|^2 . \tag{26}$$

We can express this as a matrix multiplication. Let the vector $\mathbf{X}$ be the vectorized version of all time responses; one time response is arranged to a column vector and all of them are stacked on top of each other. The DCT can be expressed as matrix multiplication, so the DCT with respect to time of this long vector is calculated by multiplying it from the left with the block diagonal matrix $\mathbf{D}$ where the main-diagonal blocks are the square DCT matrices $\mathbf{DCT}_T$ of size $T \times T$:

$$\mathbf{D} = \begin{bmatrix} \mathbf{DCT}_T & \mathbf{0} & \dots & \mathbf{0} \\ \mathbf{0} & \mathbf{DCT}_T & \dots & \mathbf{0} \\ \vdots & \vdots & \ddots & \vdots \\ \mathbf{0} & \mathbf{0} & \dots & \mathbf{DCT}_T \end{bmatrix} . \tag{27}$$

The diagonal matrix $\mathbf{C}$ contains the (repeated) values of the illumination phasor field pulse $\mathcal{P}_{\mathcal{F}}(x_{\mathrm{p}}, y_{\mathrm{p}}, 0, \Omega)$ and is multiplied to the result from the left. Convolutions can be expressed as matrix multiplication, so let $\mathbf{B}$ be the matrix that performs the 2D convolution of the values corresponding to frequency index Ω. Last but not least, matrix $\mathbf{A}$ takes care of the inverse Fourier

transform with respect to time at the corresponding time index. This results in the vector $\mathbf{Y}$ of random variables representing the brightness (before calculating the absolute value squared) of the reconstruction voxels at depth z_{v} being calculated by

$$\mathbf{Y} = \underbrace{\mathbf{ABCD}}_{=:\mathbf{M}}\mathbf{X}\,. \tag{28}$$

Elementwise calculation of the mean therefore yields

$$\mathbb{E}\{\mathbf{Y}\} = \mathbf{M}\,\mathbb{E}\{\mathbf{X}\}\,, \tag{29}$$

and the covariance matrix $\mathbf{K_{YY}}$ is calculated using (12). Note that $\mathbf{K_{XX}}$ is a diagonal matrix because the random variables in the time bins are considered independent and their pairwise covariance therefore equals zero. $\mathbf{K_{XX}}$ has the original variances $\Lambda(\mathbf{x}_{\mathrm{p}} \to \mathbf{x}_{\mathrm{c}}, t)$ on its diagonal (see (22)), and subsequently,

$$\mathbf{K_{YY}} = \mathbf{M}\mathbf{K_{XX}}\mathbf{M}^{\mathrm{H}}\,. \tag{30}$$

The variances of the resulting vector $\mathbf{Y}$ are the diagonal elements of $\mathbf{K_{YY}}$. The final question is how to calculate the mean and the variance of the absolute values squared of the elements of $\mathbf{Y}$. Note that each of these elements is a complex number, so we split $\mathbf{M}$ in its real and imaginary part $\mathbf{R} = \Re(\mathbf{M})$ and $\mathbf{S} = \Im(\mathbf{M})$, respectively.

We consider the i-th element of $\mathbf{Y}$, $\mathbf{Y}_i$, corresponding to some reconstruction voxel of interest. It is calculated by

$$\mathbf{Y}_i = \mathbf{R}_i\mathbf{X} + j\mathbf{S}_i\mathbf{X}\,, \tag{31}$$

where $\mathbf{R}_i$ is the i-th row of $\mathbf{R}$ (for $\mathbf{S}$ analogously). In the following, the SNR will be calculated.

Let us calculate the mean first. We are interested in

$$\mathbb{E}\{|\mathbf{Y}_i|^2\} = \mathbb{E}\{(\mathbf{R}_i\mathbf{X})^2 + (\mathbf{S}_i\mathbf{X})^2\} = \mathbb{E}\{(\mathbf{R}_i\mathbf{X})^2\} + \mathbb{E}\{(\mathbf{S}_i\mathbf{X})^2\}\,. \tag{32}$$

From the definition of the variance, $\mathrm{Var}\{X\} = \mathbb{E}\{X^2\} - \mathbb{E}\{X\}^2$, we see that

$$\mathbb{E}\{X^2\} = \mathbb{E}\{X\}^2 + \mathrm{Var}\{X\} \tag{33}$$

and we can reformulate (32) as

$$\begin{aligned}\mathbb{E}\{|\mathbf{Y}_i|^2\} &= (\mathbb{E}\{\mathbf{R}_i\mathbf{X}\})^2 + \mathrm{Var}\{(\mathbf{R}_i\mathbf{X})\} + (\mathbb{E}\{\mathbf{S}_i\mathbf{X}\})^2 + \mathrm{Var}\{(\mathbf{S}_i\mathbf{X})\} \\ &= (\mathbf{R}_i\overline{\mathbf{X}})^2 + (\mathbf{R}_i).^2\mathrm{Var}\{\mathbf{X}\} + (\mathbf{S}_i\overline{\mathbf{X}})^2 + (\mathbf{S}_i).^2\mathrm{Var}\{\mathbf{X}\}\,,\end{aligned} \tag{34}$$

where it is important to emphasize that the mean value of the squared variables does not only depend on the mean value squared of the original variable, but also its variance. For the simplification of the variance we have used $\mathrm{Var}\{aX + bY\} = a^2\mathrm{Var}\{X\} + b^2\mathrm{Var}\{Y\} + 2ab\mathrm{Cov}\{X, Y\}$; note that the random variables in $\mathbf{X}$ are independent and the covariances therefore are zero. $(\cdot).^2$

denotes elementwise squaring of a matrix or, as in this case, vector. $\overline{\mathbf{X}}$ and $\mathrm{Var}\{\mathbf{X}\}$ in (34) are vectors (mean and variance are calculated elementwise) and can be replaced by the corresponding $\Lambda(\mathbf{x}_\mathrm{p} \to \mathbf{x}_\mathrm{c}, t)$ from (22) (recall that for Poisson-distributed random variables, the mean equals the variance). When undergoing the same vectorization process as the time responses, we can relabel the $\Lambda(\mathbf{x}_\mathrm{p} \to \mathbf{x}_\mathrm{c}, t)$ to Λ_i.

For the variance, we can write using the standard formula $\mathrm{Var}\{X\} = \mathbb{E}\{X^2\} - \mathbb{E}\{X\}^2$

$$\begin{aligned}\mathrm{Var}\{|\mathbf{Y}_i|^2\} &= \mathrm{Var}\{\Re(\mathbf{Y}_i)^2 + \Im(\mathbf{Y}_i)^2\} \\ &= \mathbb{E}\{(\Re(\mathbf{Y}_i)^2 + \Im(\mathbf{Y}_i)^2)^2\} - \mathbb{E}\{\Re(\mathbf{Y}_i)^2 + \Im(\mathbf{Y}_i)^2\}^2 \\ &= \mathbb{E}\{\Re(\mathbf{Y}_i)^4 + 2\Re(\mathbf{Y}_i)^2\Im(\mathbf{Y}_i)^2 + \Im(\mathbf{Y}_i)^4\} - \mathbb{E}\{|\mathbf{Y}_i|^2\}^2 \\ &= \mathbb{E}\{\Re(\mathbf{Y}_i)^4\} + 2\,\mathbb{E}\{\Re(\mathbf{Y}_i)^2\Im(\mathbf{Y}_i)^2\} + \mathbb{E}\{\Im(\mathbf{Y}_i)^4\} - \mathbb{E}\{|\mathbf{Y}_i|^2\}^2 \,.\end{aligned} \tag{35}$$

The terms $\mathbb{E}\{\Re(\mathbf{Y}_i)^4\}$ and $\mathbb{E}\{\Im(\mathbf{Y}_i)^4\}$ can be calculated easily using moment-generating functions, see below. For $2\,\mathbb{E}\{\Re(\mathbf{Y}_i)^2\Im(\mathbf{Y}_i)^2\}$, one has to keep in mind that the real and the imaginary part are correlated; we can calculate the covariance matrix as in (30), but this time with a reduced matrix $\mathbf{M}_\mathrm{red}$ consisting of just the two rows representing only the real and imaginary parts of $\mathbf{M}$ belonging to one particular reconstruction voxel. We see that the resulting covariance matrix (of size 2×2) in general is not a diagonal matrix, so the real and imaginary parts are correlated and thus $\mathbb{E}\{\Re(\mathbf{Y}_i)^2\Im(\mathbf{Y}_i)^2\} \neq \mathbb{E}\{\Re(\mathbf{Y}_i)^2\}\,\mathbb{E}\{\Im(\mathbf{Y}_i)^2\}$. Let us therefore move on by writing

$$\begin{aligned}&\mathbb{E}\{\Re(\mathbf{Y}_i)^2\Im(\mathbf{Y}_i)^2\} \\ &= \mathbb{E}\{(\mathbf{R}_i\mathbf{X})^2(\mathbf{S}_i\mathbf{X})^2\} \\ &= \mathbb{E}\{\left(\sum_{k=1}^{K}\mathbf{R}_{ik}\mathbf{X}_k\right)^2\left(\sum_{k=1}^{K}\mathbf{S}_{ik}\mathbf{X}_k\right)^2\} \\ &= \mathbb{E}\{\left(\sum_{k=1}^{K}\mathbf{R}_{ik}^2\mathbf{X}_k^2 + 2\sum_{j=1}^{K}\sum_{l=1}^{j-1}\mathbf{R}_{ij}\mathbf{R}_{il}\mathbf{X}_j\mathbf{X}_l\right)\left(\sum_{k=1}^{K}\mathbf{S}_{ik}^2\mathbf{X}_k^2 + 2\sum_{j=1}^{K}\sum_{l=1}^{j-1}\mathbf{S}_{ij}\mathbf{S}_{il}\mathbf{X}_j\mathbf{X}_l\right)\} \\ &= \mathbb{E}\{\left(\sum_{k=1}^{K}\mathbf{R}_{ik}^2\mathbf{X}_k^2\right)\left(\sum_{k=1}^{K}\mathbf{S}_{ik}^2\mathbf{X}_k^2\right)\} + 2\,\mathbb{E}\{\left(\sum_{k=1}^{K}\mathbf{R}_{ik}^2\mathbf{X}_k^2\right)\left(\sum_{j=1}^{K}\sum_{l=1}^{j-1}\mathbf{S}_{ij}\mathbf{S}_{il}\mathbf{X}_j\mathbf{X}_l\right)\} \\ &\quad + 2\,\mathbb{E}\{\left(\sum_{j=1}^{K}\sum_{l=1}^{j-1}\mathbf{R}_{ij}\mathbf{R}_{il}\mathbf{X}_j\mathbf{X}_l\right)\left(\sum_{k=1}^{K}\mathbf{S}_{ik}^2\mathbf{X}_k^2\right)\} \\ &\quad + 4\,\mathbb{E}\{\left(\sum_{j=1}^{K}\sum_{l=1}^{j-1}\mathbf{R}_{ij}\mathbf{R}_{il}\mathbf{X}_j\mathbf{X}_l\right)\left(\sum_{j=1}^{K}\sum_{l=1}^{j-1}\mathbf{S}_{ij}\mathbf{S}_{il}\mathbf{X}_j\mathbf{X}_l\right)\} \,.\end{aligned} \tag{36}$$

For simplification, we made use of the formula

$$\left(\sum_{k=1}^{K}a_k\mathbf{X}_k\right)^2 = \sum_{k=1}^{K}a_k^2\mathbf{X}_k^2 + 2\sum_{j=1}^{K}\sum_{l=1}^{j-1}a_ja_l\mathbf{X}_j\mathbf{X}_l \,. \tag{37}$$

Let us now consider the individual terms of (36):

$$\begin{aligned}\mathbb{E}\{\left(\sum_{k=1}^{K}\mathbf{R}_{ik}^{2}\mathbf{X}_{k}^{2}\right)\left(\sum_{k=1}^{K}\mathbf{S}_{ik}^{2}\mathbf{X}_{k}^{2}\right)\} &= \mathbb{E}\{\sum_{k=1}^{K}\sum_{\substack{k'=1,\\k\neq k'}}^{K}\mathbf{R}_{ik}^{2}\mathbf{X}_{k}^{2}\mathbf{S}_{ik'}^{2}\mathbf{X}_{k'}^{2}\} + \mathbb{E}\{\sum_{k=1}^{K}\mathbf{R}_{ik}^{2}\mathbf{S}_{ik}^{2}\mathbf{X}_{k}^{4}\}\\ &= \sum_{k=1}^{K}\sum_{\substack{k'=1,\\k\neq k'}}^{K}\mathbf{R}_{ik}^{2}\,\mathbb{E}\{\mathbf{X}_{k}^{2}\}\mathbf{S}_{ik'}^{2}\,\mathbb{E}\{\mathbf{X}_{k'}^{2}\} + \sum_{k=1}^{K}\mathbf{R}_{ik}^{2}\mathbf{S}_{ik}^{2}\,\mathbb{E}\{\mathbf{X}_{k}^{4}\}\,,\end{aligned} \tag{38}$$

$$\begin{aligned}&\mathbb{E}\{\left(\sum_{k=1}^{K}\mathbf{R}_{ik}^{2}\mathbf{X}_{k}^{2}\right)\left(\sum_{j=1}^{K}\sum_{l=1}^{j-1}\mathbf{S}_{ij}\mathbf{S}_{il}\mathbf{X}_{j}\mathbf{X}_{l}\right)\}\\ &= \mathbb{E}\{\sum_{k=1}^{K}\sum_{\substack{j=1,\\j\neq k}}^{K}\sum_{\substack{l=1,\\l\neq k}}^{j-1}\mathbf{R}_{ik}^{2}\mathbf{X}_{k}^{2}\mathbf{S}_{ij}\mathbf{S}_{il}\mathbf{X}_{j}\mathbf{X}_{l}\} + \mathbb{E}\{\sum_{k=1}^{K}\sum_{\substack{l=1,\\l\neq k}}^{k-1}\mathbf{R}_{ik}^{2}\mathbf{X}_{k}^{3}\mathbf{S}_{ik}\mathbf{S}_{il}\mathbf{X}_{l}\} + \mathbb{E}\{\sum_{k=1}^{K}\sum_{\substack{j=1,\\j\neq k}}^{K}\mathbf{R}_{ik}^{2}\mathbf{X}_{k}^{3}\mathbf{S}_{ij}\mathbf{S}_{ik}\mathbf{X}_{j}\}\\ &= \sum_{k=1}^{K}\sum_{\substack{j=1,\\j\neq k}}^{K}\sum_{\substack{l=1,\\l\neq k}}^{j-1}\mathbf{R}_{ik}^{2}\,\mathbb{E}\{\mathbf{X}_{k}^{2}\}\mathbf{S}_{ij}\mathbf{S}_{il}\,\mathbb{E}\{\mathbf{X}_{j}\}\,\mathbb{E}\{\mathbf{X}_{l}\} + \sum_{k=1}^{K}\sum_{\substack{l=1,\\l\neq k}}^{k-1}\mathbf{R}_{ik}^{2}\,\mathbb{E}\{\mathbf{X}_{k}^{3}\}\mathbf{S}_{ik}\mathbf{S}_{il}\,\mathbb{E}\{\mathbf{X}_{l}\}\\ &\quad + \sum_{k=1}^{K}\sum_{\substack{j=1,\\j\neq k}}^{K}\mathbf{R}_{ik}^{2}\,\mathbb{E}\{\mathbf{X}_{k}^{3}\}\mathbf{S}_{ij}\mathbf{S}_{ik}\,\mathbb{E}\{\mathbf{X}_{j}\}\,,\end{aligned} \tag{39}$$

$$\begin{aligned}&\mathbb{E}\{\left(\sum_{k=1}^{K}\mathbf{S}_{ik}^{2}\mathbf{X}_{k}^{2}\right)\left(\sum_{j=1}^{K}\sum_{l=1}^{j-1}\mathbf{R}_{ij}\mathbf{R}_{il}\mathbf{X}_{j}\mathbf{X}_{l}\right)\}\\ &= \mathbb{E}\{\sum_{k=1}^{K}\sum_{\substack{j=1,\\j\neq k}}^{K}\sum_{\substack{l=1,\\l\neq k}}^{j-1}\mathbf{S}_{ik}^{2}\mathbf{X}_{k}^{2}\mathbf{R}_{ij}\mathbf{R}_{il}\mathbf{X}_{j}\mathbf{X}_{l}\} + \mathbb{E}\{\sum_{k=1}^{K}\sum_{\substack{l=1,\\l\neq k}}^{k-1}\mathbf{S}_{ik}^{2}\mathbf{X}_{k}^{3}\mathbf{R}_{ik}\mathbf{R}_{il}\mathbf{X}_{l}\} + \mathbb{E}\{\sum_{k=1}^{K}\sum_{\substack{j=1,\\j\neq k}}^{K}\mathbf{S}_{ik}^{2}\mathbf{X}_{k}^{3}\mathbf{R}_{ij}\mathbf{R}_{ik}\mathbf{X}_{j}\}\\ &= \sum_{k=1}^{K}\sum_{\substack{j=1,\\j\neq k}}^{K}\sum_{\substack{l=1,\\l\neq k}}^{j-1}\mathbf{S}_{ik}^{2}\,\mathbb{E}\{\mathbf{X}_{k}^{2}\}\mathbf{R}_{ij}\mathbf{R}_{il}\,\mathbb{E}\{\mathbf{X}_{j}\}\,\mathbb{E}\{\mathbf{X}_{l}\} + \sum_{k=1}^{K}\sum_{\substack{l=1,\\l\neq k}}^{k-1}\mathbf{S}_{ik}^{2}\,\mathbb{E}\{\mathbf{X}_{k}^{3}\}\mathbf{R}_{ik}\mathbf{R}_{il}\,\mathbb{E}\{\mathbf{X}_{l}\}\\ &\quad + \sum_{k=1}^{K}\sum_{\substack{j=1,\\j\neq k}}^{K}\mathbf{S}_{ik}^{2}\,\mathbb{E}\{\mathbf{X}_{k}^{3}\}\mathbf{R}_{ij}\mathbf{R}_{ik}\,\mathbb{E}\{\mathbf{X}_{j}\}\,,\end{aligned} \tag{40}$$

$$
\begin{aligned}
&\mathbb{E}\{\left(\sum_{j=1}^{K}\sum_{l=1}^{j-1}\mathbf{R}_{ij}\mathbf{R}_{il}\mathbf{X}_j\mathbf{X}_l\right)\left(\sum_{j=1}^{K}\sum_{l=1}^{j-1}\mathbf{S}_{ij}\mathbf{S}_{il}\mathbf{X}_j\mathbf{X}_l\right)\} = \mathbb{E}\{\sum_{j=1}^{K}\sum_{l=1}^{j-1}\sum_{j'=1}^{K}\sum_{l'=1}^{j'-1}\mathbf{R}_{ij}\mathbf{R}_{il}\mathbf{X}_j\mathbf{X}_l\mathbf{S}_{ij'}\mathbf{S}_{il'}\mathbf{X}_{j'}\mathbf{X}_{l'}\} \\
&= \mathbb{E}\{\sum_{j=1}^{K}\sum_{l=1}^{j-1}\sum_{\substack{j'=1,\\ j'\neq j,\\ j'\neq l}}^{K}\sum_{\substack{l'=1,\\ l'\neq j,\\ l'\neq l}}^{j'-1}\mathbf{R}_{ij}\mathbf{R}_{il}\mathbf{X}_j\mathbf{X}_l\mathbf{S}_{ij'}\mathbf{S}_{il'}\mathbf{X}_{j'}\mathbf{X}_{l'}\} + \mathbb{E}\{\sum_{j=1}^{K}\sum_{l=1}^{j-1}\sum_{\substack{l'=1,\\ l'\neq j,\\ l'\neq l}}^{j-1}\mathbf{R}_{ij}\mathbf{R}_{il}\mathbf{X}_j^2\mathbf{X}_l\mathbf{S}_{ij}\mathbf{S}_{il'}\mathbf{X}_{l'}\} \\
&\quad + \mathbb{E}\{\sum_{j=1}^{K}\sum_{l=1}^{j-1}\sum_{\substack{l'=1,\\ l'\neq j,\\ l'\neq l}}^{j'-1}\mathbf{R}_{ij}\mathbf{R}_{il}\mathbf{X}_j\mathbf{X}_l^2\mathbf{S}_{il}\mathbf{S}_{il'}\mathbf{X}_{l'}\} + \mathbb{E}\{\sum_{j=1}^{K}\sum_{l=1}^{j-1}\sum_{\substack{j'=1,\\ j'\neq j,\\ j'\neq l}}^{K}\mathbf{R}_{ij}\mathbf{R}_{il}\mathbf{X}_j^2\mathbf{X}_l\mathbf{S}_{ij'}\mathbf{S}_{ij}\mathbf{X}_{j'}\} \\
&\quad + \mathbb{E}\{\sum_{j=1}^{K}\sum_{l=1}^{j-1}\sum_{\substack{j'=1,\\ j'\neq j,\\ j'\neq l}}^{K}\mathbf{R}_{ij}\mathbf{R}_{il}\mathbf{X}_j\mathbf{X}_l^2\mathbf{S}_{ij'}\mathbf{S}_{il}\mathbf{X}_{j'}\} \\
&= \sum_{j=1}^{K}\sum_{l=1}^{j-1}\sum_{\substack{j'=1,\\ j'\neq j,\\ j'\neq l}}^{K}\sum_{\substack{l'=1,\\ l'\neq j,\\ l'\neq l}}^{j'-1}\mathbf{R}_{ij}\mathbf{R}_{il}\,\mathbb{E}\{\mathbf{X}_j\}\,\mathbb{E}\{\mathbf{X}_l\}\mathbf{S}_{ij'}\mathbf{S}_{il'}\,\mathbb{E}\{\mathbf{X}_{j'}\}\,\mathbb{E}\{\mathbf{X}_{l'}\} \\
&\quad + \sum_{j=1}^{K}\sum_{l=1}^{j-1}\sum_{\substack{l'=1,\\ l'\neq j,\\ l'\neq l}}^{j-1}\mathbf{R}_{ij}\mathbf{R}_{il}\,\mathbb{E}\{\mathbf{X}_j^2\}\,\mathbb{E}\{\mathbf{X}_l\}\mathbf{S}_{ij}\mathbf{S}_{il'}\,\mathbb{E}\{\mathbf{X}_{l'}\} \\
&\quad + \sum_{j=1}^{K}\sum_{l=1}^{j-1}\sum_{\substack{l'=1,\\ l'\neq j,\\ l'\neq l}}^{j'-1}\mathbf{R}_{ij}\mathbf{R}_{il}\,\mathbb{E}\{\mathbf{X}_j\}\,\mathbb{E}\{\mathbf{X}_l^2\}\mathbf{S}_{il}\mathbf{S}_{il'}\,\mathbb{E}\{\mathbf{X}_{l'}\} + \sum_{j=1}^{K}\sum_{l=1}^{j-1}\sum_{\substack{j'=1,\\ j'\neq j,\\ j'\neq l}}^{K}\mathbf{R}_{ij}\mathbf{R}_{il}\,\mathbb{E}\{\mathbf{X}_j^2\}\,\mathbb{E}\{\mathbf{X}_l\}\mathbf{S}_{ij'}\mathbf{S}_{ij}\,\mathbb{E}\{\mathbf{X}_{j'}\} \\
&\quad + \sum_{j=1}^{K}\sum_{l=1}^{j-1}\sum_{\substack{j'=1,\\ j'\neq j,\\ j'\neq l}}^{K}\mathbf{R}_{ij}\mathbf{R}_{il}\,\mathbb{E}\{\mathbf{X}_j\}\,\mathbb{E}\{\mathbf{X}_l^2\}\mathbf{S}_{ij'}\mathbf{S}_{il}\,\mathbb{E}\{\mathbf{X}_{j'}\}\,.
\end{aligned}
\tag{41}
$$

The moment-generating function of a Poisson-distributed variable with parameter Λ is defined by

$$M_X(s) = e^{\Lambda(e^s-1)}\,, \tag{42}$$

and for a linear combination of independent Poisson-distributed random variables we get using (14)

$$M_X(s) = e^{\sum_k^K \Lambda_k(e^{a_k s}-1)}\,. \tag{43}$$

Equation (36) has to be evaluated carefully. We have to calculate the expected values of the product of four different random variables, e.g., $\mathbb{E}\{\mathbf{X}_1\mathbf{X}_2\mathbf{X}_3\mathbf{X}_4\}$, and the fourth moment of a random variable $\mathbb{E}\{\mathbf{X}_1^4\}$, and all combinations in between. A fortunate factor is that these random variables are independent (they are the original Poisson-distributed ones), so we can write

$\mathbb{E}\{\mathbf{X}_1\mathbf{X}_2\mathbf{X}_3\mathbf{X}_4\} = \mathbb{E}\{\mathbf{X}_1\}\,\mathbb{E}\{\mathbf{X}_2\}\,\mathbb{E}\{\mathbf{X}_3\}\,\mathbb{E}\{\mathbf{X}_4\}$, but we have to be careful when there are terms like $\mathbb{E}\{\mathbf{X}_1\mathbf{X}_1\mathbf{X}_1\mathbf{X}_1\}$, which has to be evaluated as $\mathbb{E}\{\mathbf{X}_1^4\}$ and not $\mathbb{E}\{\mathbf{X}_1\}\,\mathbb{E}\{\mathbf{X}_1\}\,\mathbb{E}\{\mathbf{X}_1\}\,\mathbb{E}\{\mathbf{X}_1\}$ (such terms appear for example in $\left(\sum_{k=1}^K \mathbf{R}_{ik}^2\mathbf{X}_k^2\right)\left(\sum_{k=1}^K \mathbf{S}_{ik}^2\mathbf{X}_k^2\right)$). For our Poisson-distributed random variables we have

$$\begin{aligned}\mathbb{E}\{\mathbf{X}_k\} &= \Lambda_k\\ \mathbb{E}\{\mathbf{X}_k^2\} &= \Lambda_k^2 + \Lambda_k\\ \mathbb{E}\{\mathbf{X}_k^3\} &= \Lambda_k^3 + 3\Lambda_k^2 + \Lambda_k\\ \mathbb{E}\{\mathbf{X}_k^4\} &= \Lambda_k^4 + 6\Lambda_k^3 + 7\Lambda_k^2 + \Lambda_k\,.\end{aligned} \tag{44}$$

Applying Mathematica to (43) for calculating the term $\mathbb{E}\{\Re(\mathbf{Y}_i)^4\} = \mathbb{E}\{(\mathbf{R}_i\mathbf{X})^4\}$ in (35) (note that unlike the single X_k, this is a linear combination of all of these Poisson-distributed random variables) yields

$$\begin{aligned}\mathbb{E}\{(\mathbf{R}_i\mathbf{X})^4\} = &\left(\sum_{k=1}^K \mathbf{R}_{ik}\Lambda_k\right)^4 + 6\left(\sum_{k=1}^K \mathbf{R}_{ik}\Lambda_k\right)^2\left(\sum_{k=1}^K \mathbf{R}_{ik}^2\Lambda_k\right) + 3\left(\sum_{k=1}^K \mathbf{R}_{ik}^2\Lambda_k\right)^2\\ &+ 4\left(\sum_{k=1}^K \mathbf{R}_{ik}\Lambda_k\right)\left(\sum_{k=1}^K \mathbf{R}_{ik}^3\Lambda_k\right) + \left(\sum_{k=1}^K \mathbf{R}_{ik}^4\Lambda_k\right)\,.\end{aligned} \tag{45}$$

$\mathbb{E}\{\Im(\mathbf{Y}_i)^4\} = \mathbb{E}\{(\mathbf{S}_i\mathbf{X})^4\}$ is calculated analogously.

After having calculated the general SNR according to Eq. (9) for an arbitrary reconstruction voxel, it is hard to tell what exactly the SNR curve as a function of the distance z_v between the reconstruction voxel and the relay wall looks like. Even one row vector of the matrix M has hundreds of thousands of elements, and it is hard to evaluate their effect analytically. However, what we can do is to consider the full SNR derived in the case of a large distance z_v, first in the squared case. The parameters Λ_k denote the means and the variances of the original Poisson variables in each time bin; for large distances $z_\mathrm{v} \approx \sqrt{(x_\mathrm{v}-x_\mathrm{p})^2+(y_\mathrm{v}-y_\mathrm{p})^2+z_\mathrm{v}^2} \approx \sqrt{(x_\mathrm{v}-x_\mathrm{c})^2+(y_\mathrm{v}-y_\mathrm{c})^2+z_\mathrm{v}^2}$ they are approximated by $1/z_\mathrm{v}^4$. The mean value from Eq. (34) has terms of the form $a/z_\mathrm{v}^4 + b/z_\mathrm{v}^8$ (variance of the original random variables goes with $1/z_\mathrm{v}^4$, plus their mean squared which follows $1/z_\mathrm{v}^8$), where we can neglect the higher order exponent. For the variance, we have terms of the order $1/z_\mathrm{v}^4$ (last terms of Eq. (44) and Eq. (45)), where the Poisson means Λ_k and therefore the attenuation factors $\gamma(\mathbf{x}_\mathrm{p},\mathbf{x}_\mathrm{c},\mathbf{x}_\mathrm{v})$ from Eq. (20) appear linearly. The higher exponents of the attenuation factor can be ignored because $1/z_\mathrm{v}^8$, $1/z_\mathrm{v}^{12}$ and $1/z_\mathrm{v}^{16}$ fall off much faster than $1/z_\mathrm{v}^4$. For these reasons, the SNR can be approximated by

$$\mathrm{SNR}_i \propto \frac{\frac{1}{z_\mathrm{v}^4}}{\sqrt{\frac{1}{z_\mathrm{v}^4}}} = \frac{\frac{1}{z_\mathrm{v}^4}}{\frac{1}{z_\mathrm{v}^2}} = \frac{1}{z_\mathrm{v}^2}\,. \tag{46}$$

This is quite an interesting result; while all NLOS imaging publications correctly state that the signal roughly falls of with $1/z_\mathrm{v}^4$ at large distances, the variance falls off with $1/z_\mathrm{v}^2$, which cancels part of this effect! In total, this means that the SNR only falls off with $1/z_\mathrm{v}^2$.

3.4.1 Incorporating background noise

When background noise (ambient light + detector noise) is present, we add a Poisson distribution with constant mean to all histogram time bins, which means that the Poisson variables in each time bin (see Eq. (22) with the attenuation factors Eq. (20)) now consist of a component with $1/z_{\mathrm{v}}^4$ and a constant component. For calculating the SNR as the mean over the standard deviation, we have to be careful, because the mean value of the noise should not be considered. For this reason, we take the mean value of the noise-free model and calculate the variance as previously according to Eq. (35). However, when noise is present, the variance now again has terms that fall with $1/z_{\mathrm{v}}^4$, $1/z_{\mathrm{v}}^8$, $1/z_{\mathrm{v}}^{12}$ and $1/z_{\mathrm{v}}^{16}$, but also a distance-independent constant. This means that for large distances z_{v}, the standard deviation becomes a constant. In contrast to Eq. (46), the standard deviation does not balance part of the $1/z_{\mathrm{v}}^4$ fall-off of the mean, so for a constant $c_1 \gg \frac{1}{z_{\mathrm{v}}^4}$ we arrive at

$$\mathrm{SNR}_i \propto \frac{\frac{1}{z_{\mathrm{v}}^4}}{\sqrt{\frac{1}{z_{\mathrm{v}}^4} + c_1}} \approx \frac{\frac{1}{z_{\mathrm{v}}^4}}{\sqrt{c_1}} \quad \Rightarrow \quad \mathrm{SNR}_i \propto \frac{1}{z_{\mathrm{v}}^4}\,. \tag{47}$$

This means that unlike the no noise case, the SNR will eventually not follow a $\frac{1}{z_{\mathrm{v}}^2}$, but a steeper $\frac{1}{z_{\mathrm{v}}^4}$ fall-off. This is because the standard deviation will level off at some point. This point depends on the noise mean; the larger it is, the earlier the standard deviation levels off.

3.5 SNR of arbitrary linear NLOS reconstruction

Let us also briefly consider what the SNR looks like when considering a reconstruction method (for example simple backprojection) that does not require the calculation of the absolute value squared, but only the matrix multiplication from Eq. (28). In this case, the Poisson means/variances Λ_k also appear linearly in the reconstruction voxel formulas for both mean and variance, and we get the same SNR as in Eq. (46) for large distances without considering detector noise or ambient light, and the same SNR as in Eq. (47) when these degrading effects are present.

3.6 SNR Plots

Figure 3 in the main paper shows the depth-dependent SNR values. A diffuse (Lambertian reflection) square target of size 1.5 m $\times$ 1.5 m is placed at different distances from the relay wall. The relay wall is of size 1.9 m $\times$ 1.9 m with 1 cm horizontal and vertical spacing between laser points, while a single SPAD looks at the relay wall center. Up until the maximum distance available in the lab, experimental data are included as well. For the simulated data points, the time resolution is 8 ps and the Phasor field reconstruction wavelength is 8 cm. We see that at large distances, as

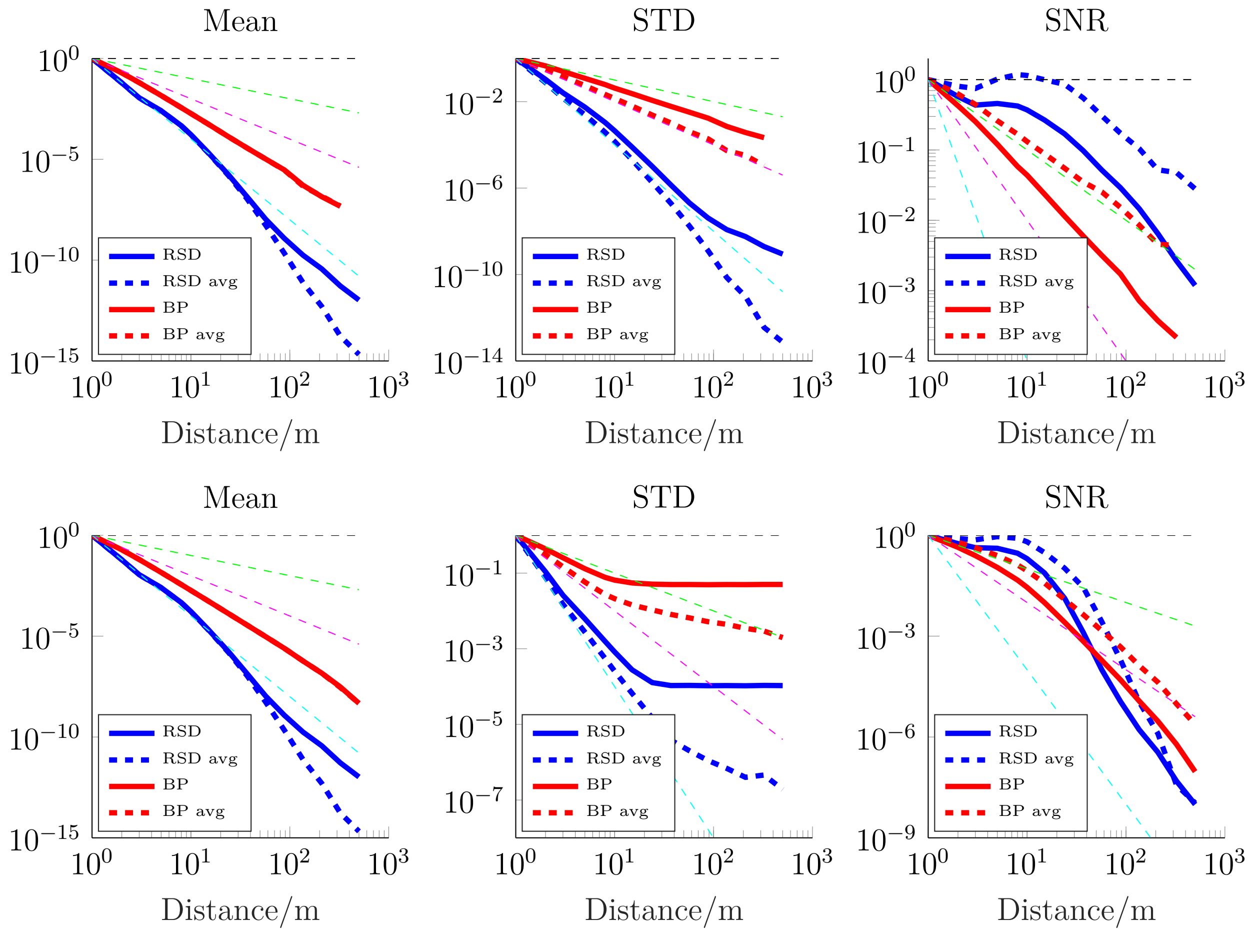

Supplementary Figure 4: Mean, standard deviation and SNR of voxels reconstructed at different depths. All curves are normalized to 1 at the closest distance. Top row: without background noise (ambient light, sensor dark count). Bottom row: with background noise. When background noise is considered, the trend derived from the theory is apparent: the standard deviation levels off at a certain distance, meaning that it doesn't cancel part of the decreasing mean anymore and the SNR falls faster after this point. Also, as expected, the standard deviation is smaller when averaging is applied, leading to a higher SNR. For each depth, the considered lateral coordinates x_v, y_v are the same. The four dashed lines are for comparison with 1 (- - -), $1/r$ (- - -), $1/r^2$ (- - -) and $1/r^4$ (- - -).

predicted in the calculation from the previous section, the SNR curve exhibits a $1/r^2$ decrease when no background noise (ambient light or sensor noise) is present. In case there is background noise (bottom plot of Fig. 4 in the main paper), the standard deviation at a specific distance levels out, and the SNR then falls with $1/r^4$. The background noise of our experimental setup is so low that this turning point is further away than the maximum possible physical distance we could realize. All shown mean and standard deviation curves are obtained by simulating the ground truth scene with the acquisition parameters (laser power, SPAD sensing area, target size and average background noise level) the same as in the real experiments. The relay wall and target are assumed to exhibit perfect diffuse (Lambertian) reflectance. Reconstructions from 20 000 different noise realizations were calculated and the sample mean and variance were calculated from the reconstructions. While Figure 4 in the main paper only shows the resulting SNR, Supplementary Fig. 4 for the sake of completeness also shows the mean and standard deviation. We see that at large distances, coinciding with the derived theory, the RSD mean falls parallel to $1/r^4$ and its standard deviation with $1/r^2$, meaning that the SNR also exhibits a $1/r^2$ characteristic. Once background noise is considered in the simulations, the standard deviation levels off at a certain distance, leading to the SNR falling steeper in a $1/r^4$ manner.

In addition, we want to provide a detailed comparison of RSD and BP SNR when different powers of the results are taken. According to (26) and (32), the RSD at the very end requires the calculation of the absolute value squared, while BP is just the linear matrix multiplication of the form (28). In the main paper, the SNR curves according to the respective power are shown. For fairness, we also show the SNR curves when the square root of the RSD result is taken and when the BP output is squared in Supplementary Fig. 5. As can be seen, the SNR curves look very similar for both RSD and BP, regardless of which power is used eventually. For BP, the mean falls roughly with $1/r^3$ and the variance with $1/r^{1.5}$, meaning that the SNR also follows a $1/r^{1.5}$ curve. Squaring the pixelwise BP results results in a mean that first falls steeper than $1/r^4$, but then changes to a falloff with $1/r^2$. This can be explained with Eq. (33); overall, we can write this approximately as

$$\mathbb{E}\{X^2\} = \mathbb{E}\{X\}^2 + \mathrm{Var}\{X\} \tag{48}$$

$$\approx \left(A\frac{1}{r^4}\right)^2 + B\frac{1}{r^4} \tag{49}$$

$$= A^2\frac{1}{r^8} + B\frac{1}{r^4} \tag{50}$$

with two constants $A, B \gg 1$, and where we assume that they only weakly depend on r and we can neglect this dependence for simplicity. This means that for small r, the first term is dominant because of the squared constant, and with increasing r, at a certain value, the second one becomes dominant. A similar argument can be made for the variance, and both these arguments together explain the curve shape for mean and standard deviation and experimentally confirm the theory derived above, albeit the distance of the turning points is much larger than the distances at which experimental results have been demonstrated in the NLOS literature so far. We want to empha-

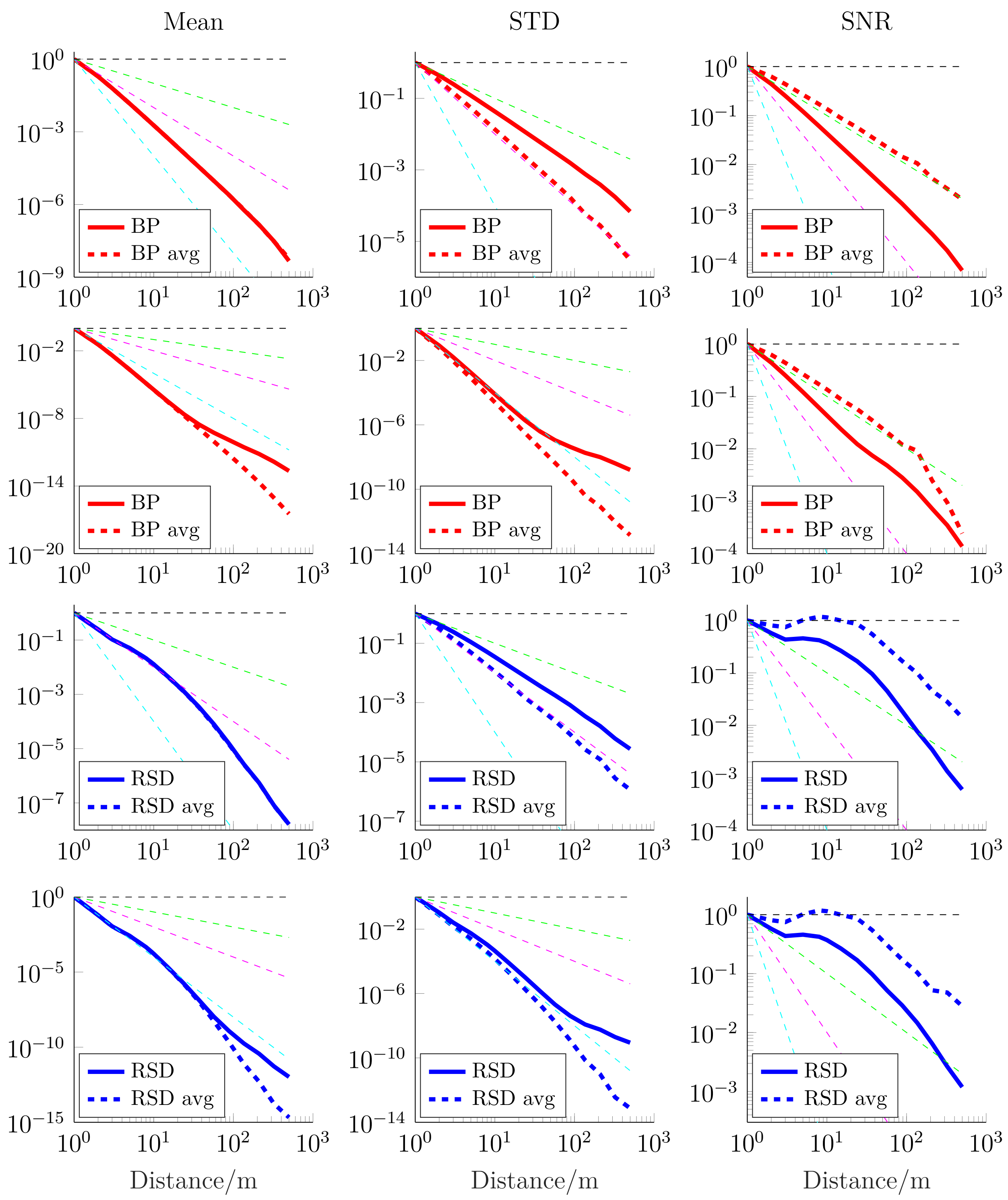

Supplementary Figure 5: Comparison of RSD and BP for different power values. All curves are normalized to 1 at the closest distance. First row: BP, power 1, second row: BP, power 2; third row: RSD, power 1, last row: RSD, power 2. The four dashed lines are for comparison with 1 (- - -), $1/r$ (- - -), $1/r^2$ (- - -) and $1/r^4$ (- - -).

size once again that the choice of the final power has practically no influence on the shape and magnitude of the SNR curves.

4 Supplementary Results

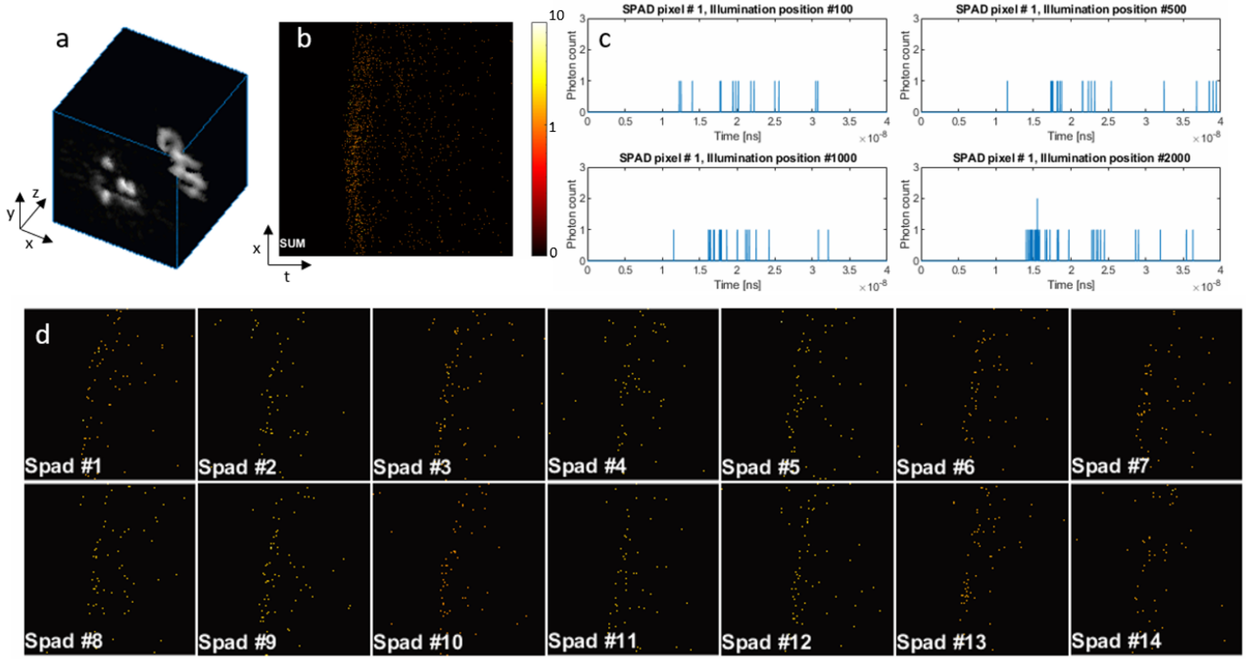

Supplementary Figure 6: **Raw data visualisation.** a) Single frame 19 of a video reconstruction. b) Virtually remapped input data acquired by 28 SPAD pixels. c) Raw data for 4 different illumination positions for SPAD pixel 1. d) Raw data for all positions for SPAD pixels 1-14.

Supplementary Figure 6 shows one frame of a live video data collected using our NLOS imaging system. Exposure time is 0.2 s. Supplementary Figure 6a displays one frame of the reconstructed video. Supplementary Figure 6b shows a spatio-temporal image of virtually remapped data from all 28 SPAD pixels. The horizontal axis is the time dimension and the vertical axis corresponds to spatial illumination positions. The color map is in log scale. Supplementary Figure 6c shows time responses for 4 different illumination positions collected by SPAD pixel 1. Lastly, Supplementary Figure 6d shows spatio-temporal images of time responses for 14 different SPAD pixels.

Additionally, we show that one can use the SPAD array and full laser grid to perform summation of output reconstructions from all SPAD pixels, see Supplementary Figure 7. The illumination wall size is 1.5 m x 1.5 m with 128x128 sampling points and 14 SPAD pixels. The exposure time is 1 s. We performed 14 separate Phasor Field reconstructions, one for each SPAD array pixel. Results are shown in Supplementary Figure 7 first and second row. The next four rows show the

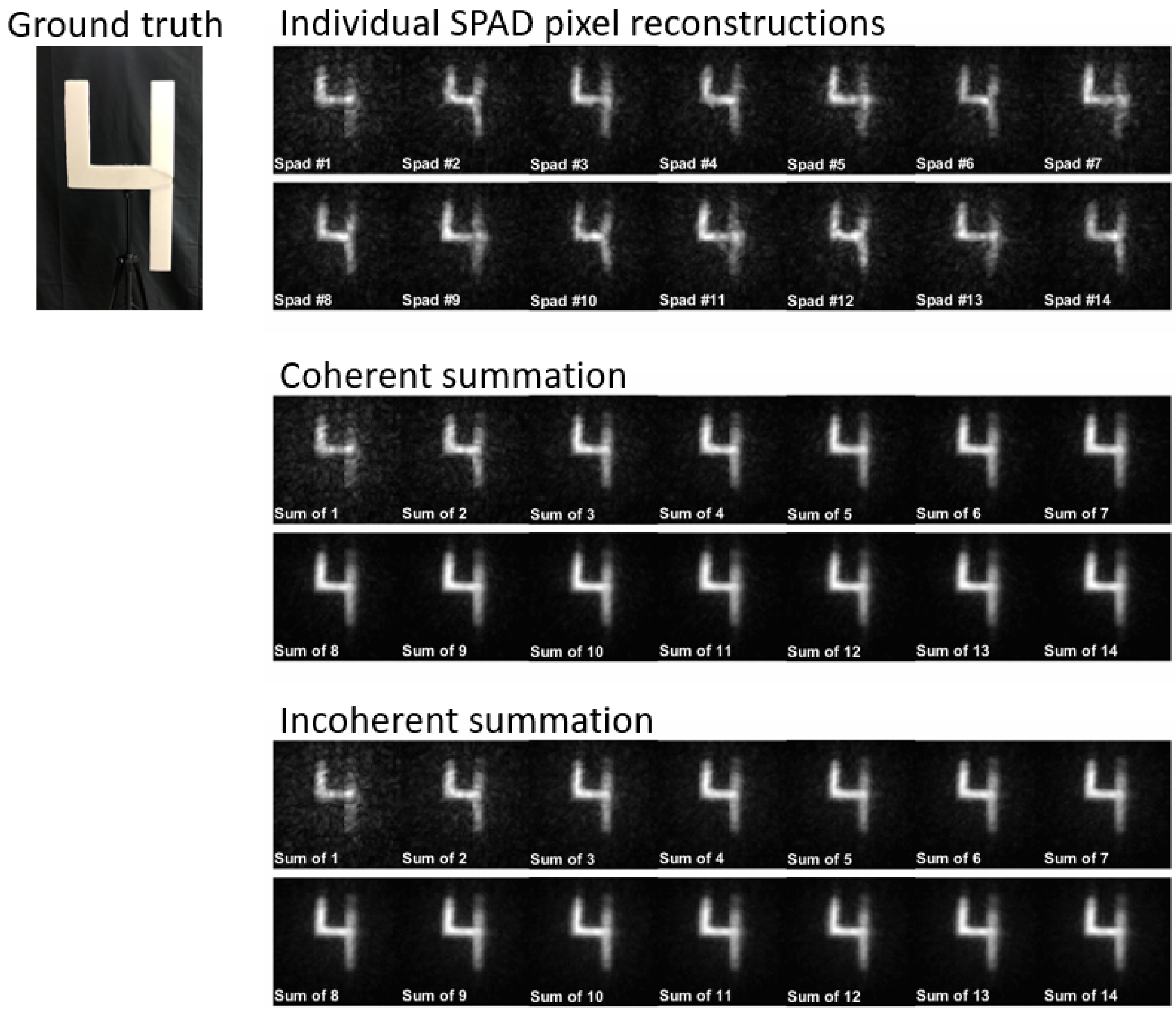

Supplementary Figure 7: **Individual SPAD pixel reconstructions and effect of coherent and incoherent summation of multiple reconstructions.**

effect of coherent and incoherent summation of multiple reconstructions. This example shows how reconstruction changes when we use multiple pixels.

Lastly, we show the effect of accumulative exposure time on stationary scenes. 100 frames were collected with an exposure time of 0.2 s per frame. Supplementary Figure 8 shows reconstructions of 3 different stationary scene with different exposure times ranging from 0.2 seconds up to 20 seconds. One can notice that the reconstructions don't change significantly after $1.4-2$ seconds of exposure time. We want to emphasize that the noise can be greatly reduced in the future by using SPAD arrays with more pixels that allow for the acquisition of more photons in the same amount of time.

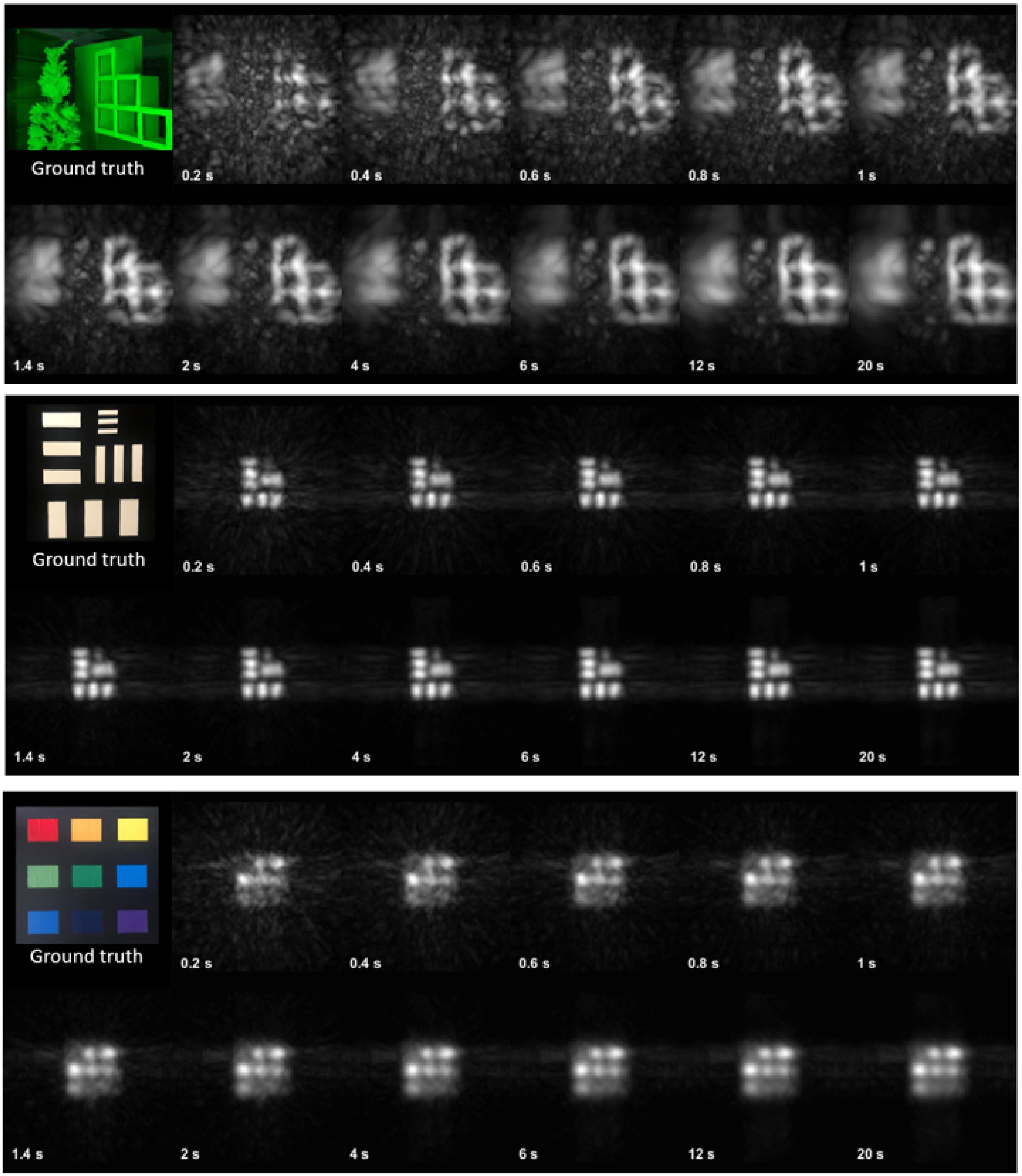

Supplementary Figure 8: **Effect of accumulative exposure time on stationary scenes.** Three different stationary scenes: 1) shelf with white Christmas tree and large wall; 2) resolution bar; 3) color bar.

5 List of used symbols

Symbol	Definition
P	Projection plane on relay wall surface
C	Camera plane on relay wall surface
$\mathbf{x}_{\mathrm{p}} = (x_{\mathrm{p}}, y_{\mathrm{p}}, 0)$	Projection point in plane P
$\mathbf{x}_{\mathrm{c}} = (x_{\mathrm{c}}, y_{\mathrm{c}}, 0)$	Camera point in plane C
$\mathbf{x}_{\mathrm{v}} = (x_{\mathrm{v}}, y_{\mathrm{v}}, z_{\mathrm{v}})$	Reconstruction voxel position
r	Shortest distance between relay surface and object, mostly used interchangably with z_{v}
t	Time
Δx_{c}	Aperture sampling distance along x
Q	Aperture scanning reduction factor
τ	Time index in the context of averaging
Ω	Frequency
Ω_{C}	Phasor field modulation center frequency
$\Delta\Omega$	Phasor field modulation frequency range
$H(\mathbf{x}_{\mathrm{p}} \to \mathbf{x}_{\mathrm{c}}, t)$	Time response at illumination point $\mathbf{x}_{\mathrm{p}}$ and acquisition point $\mathbf{x}_{\mathrm{c}}$
$\mathcal{P}_{\mathcal{F}}(\mathbf{x}_{\mathrm{p}}, \Omega)$	Frequency domain phasor field illumination pulse at point $\mathbf{x}_{\mathrm{p}}$
$\mathcal{P}_{\mathcal{F}}(\mathbf{x}_{\mathrm{c}}, \Omega)$	Frequency domain phasor field response at point $\mathbf{x}_{\mathrm{c}}$
$\mathcal{I}(\mathbf{x}_{\mathrm{v}}, t)$	Phasor field reconstruction intensity; evaluating at corresponding wave propagation time t for each pixel yields stationary reconstruction $\mathcal{I}(\mathbf{x}_{\mathrm{v}})$
$G(x_{\mathrm{c}}, y_{\mathrm{c}}, z_{\mathrm{v}}, \Omega)$	RSD convolution kernel
$\Phi(\cdot)$	Wave propagation operator
$\mathcal{V}(\tau)$	Temporal sequence of stationary reconstructions $\mathcal{I}(\mathbf{x}_{\mathrm{v}})$ indexed with τ
$\mathbb{E}\{\mathbf{X}\}$	Elementwise expected value of random vector $\mathbf{X}$
$\mathrm{Var}\{\mathbf{X}\}$	Elementwise variance of random vector $\mathbf{X}$
$\mathbf{K}_{\mathbf{XX}}$	Covariance matrix of random vector $\mathbf{X}$
$M_X(t)$	Moment generating function of random variable X
λ	Expected photon rate, i.e., number of photons per time interval
$\Lambda = \lambda \Delta t$	Expected value of photons during time interval Δt
μ	Expected value of random variable
σ	Standard deviation of random variable
$\gamma(\mathbf{x}_{\mathrm{p}}, \mathbf{x}_{\mathrm{c}}, \mathbf{x}_{\mathrm{v}})$	Light attenuation factor in dependence of positions
$\gamma(t)$	Equivalently, light attenuation factor in dependence of round trip time t
d_1, d_2, d_3, d_4	Distances: physical laser to $\mathbf{x}_{\mathrm{p}}$, $\mathbf{x}_{\mathrm{p}}$ to $\mathbf{x}_{\mathrm{v}}$, $\mathbf{x}_{\mathrm{v}}$ to $\mathbf{x}_{\mathrm{c}}$, $\mathbf{x}_{\mathrm{c}}$ to physical SPAD position
α	Relay wall attenuation factor

β	Possibly location dependent object attenuation factor
$W(t)$	Set of all points $\mathbf{x}_\mathrm{v}$ that lie on a spheroid with foci $\mathbf{x}_\mathrm{p}$ and $\mathbf{x}_\mathrm{c}$ and travel time t
$\Lambda(\mathbf{x}_\mathrm{p}, \mathbf{x}_\mathrm{c}, \mathbf{x}_\mathrm{v})$	Poisson distribution parameter
$\mathbf{M}$	Reconstruction matrix
$\mathbf{R}$, $\mathbf{S}$	Real and imaginary parts of $\mathbf{M}$
Q	Number of SPAD pixels in array
$\mathrm{PSF}(x, y, z)$	3D NLOS point spread function
ϕ, θ, r	Angular and depth parameters of $\mathrm{PSF}(x, y, z)$, $\Delta\phi$, $\Delta\theta$, Δr are the widths along each dimension
N	Number of averaged frames
I	Optical intensity
I_sr	Intensity per steradian

Supplementary Table 1: List of used symbols. In general, frequency domain quantities are denoted by an additional subscript $_\mathcal{F}$.

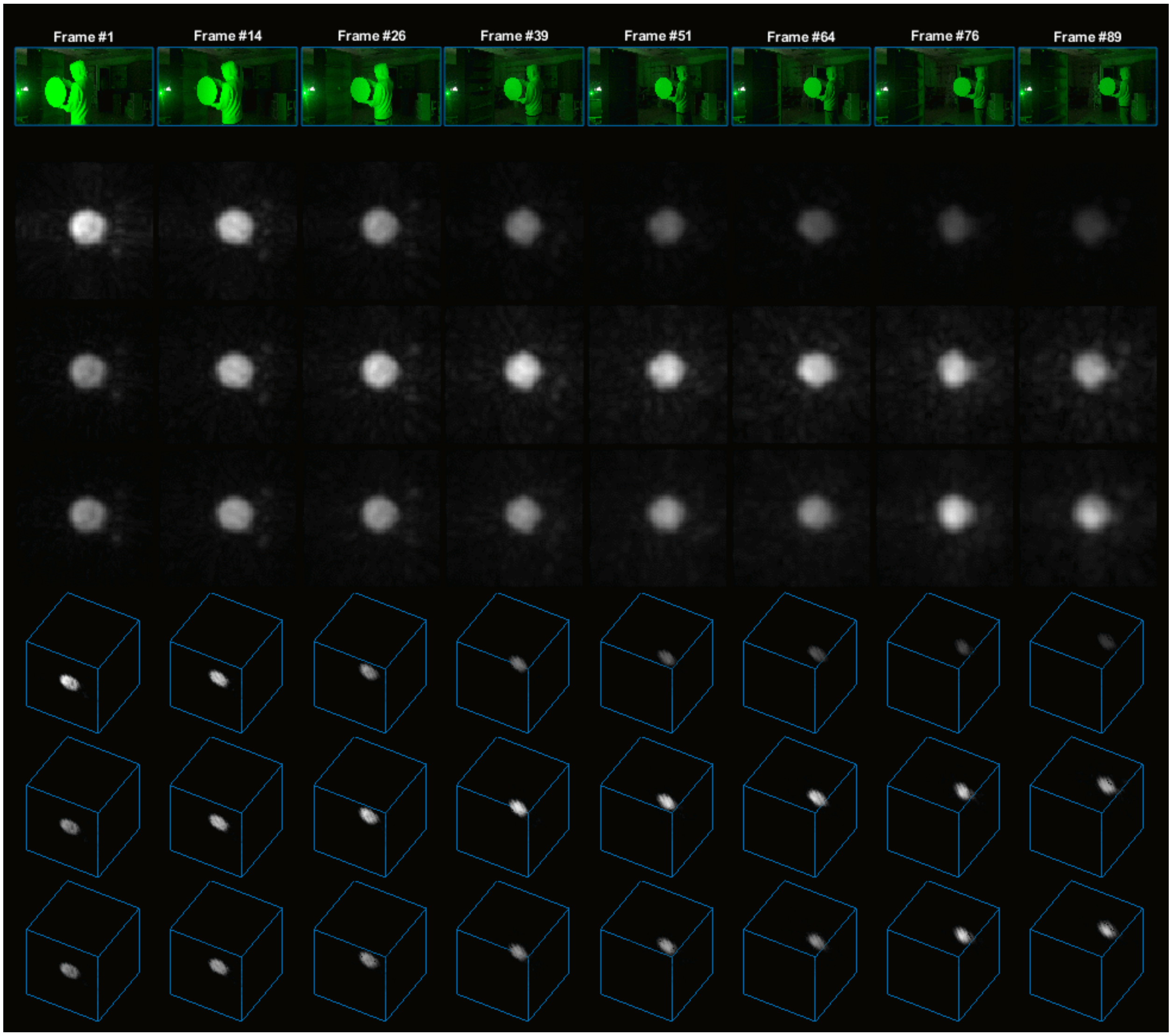

Supplementary Figure 9: **Video: “Circle”.** Ground truth, RSD, RSD with intensity correction, RSD with intensity correction and averaging.

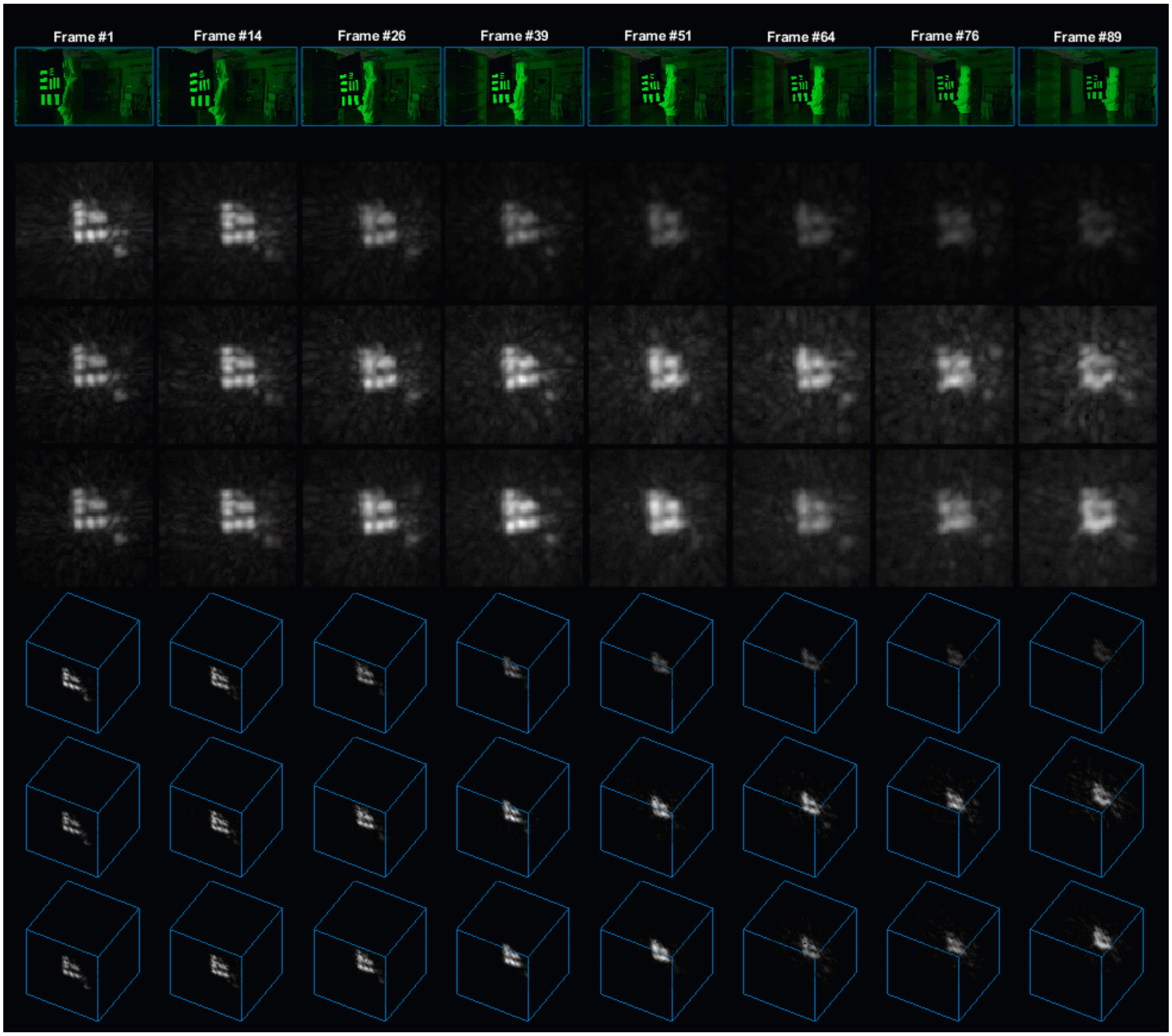

Supplementary Figure 10: **Video: "Resolution bar".** Ground truth, RSD, RSD with intensity correction, RSD with intensity correction and averaging.

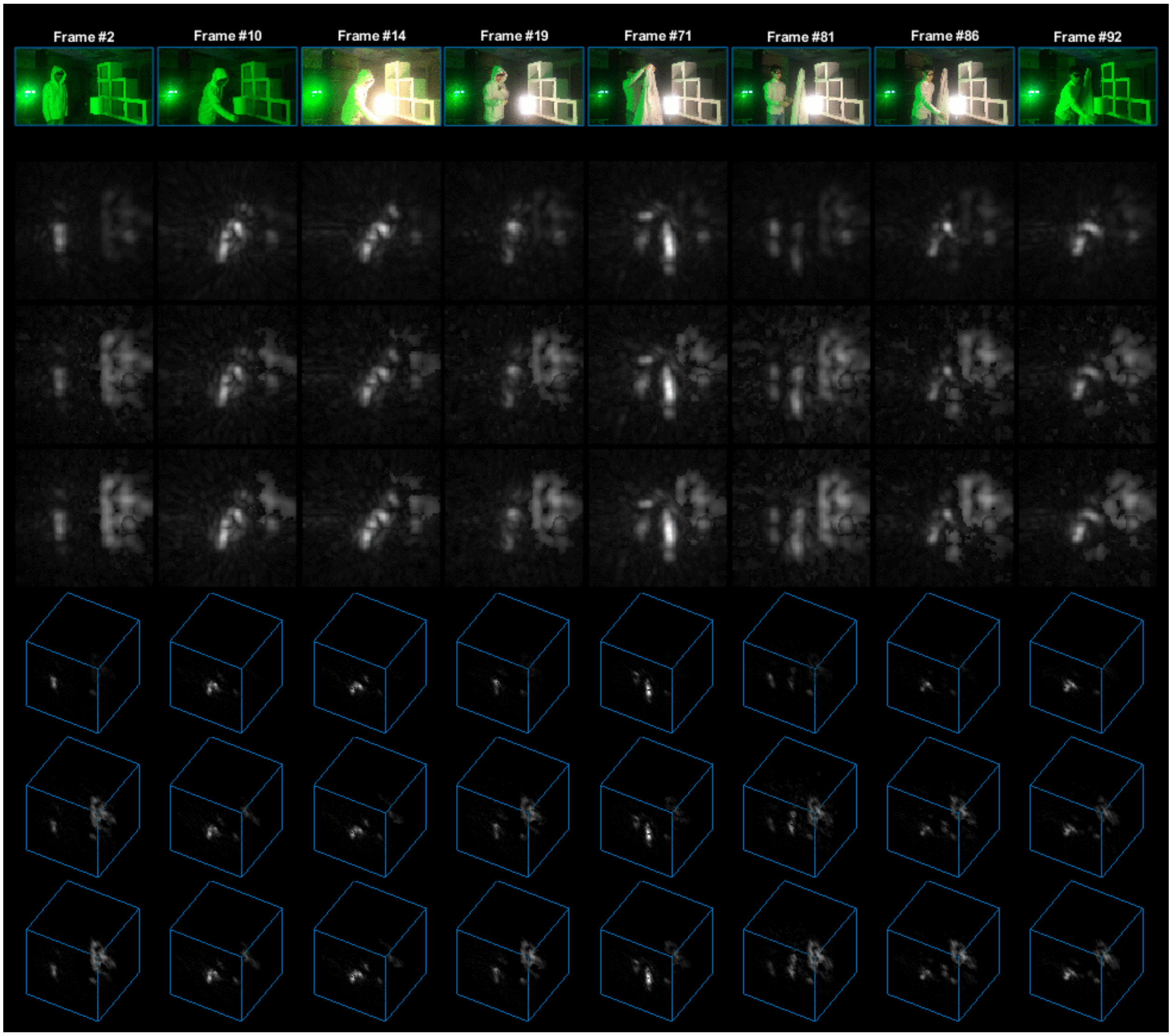

Supplementary Figure 11: **Video: "At home".** Ground truth, RSD, RSD with intensity correction, RSD with intensity correction and averaging.

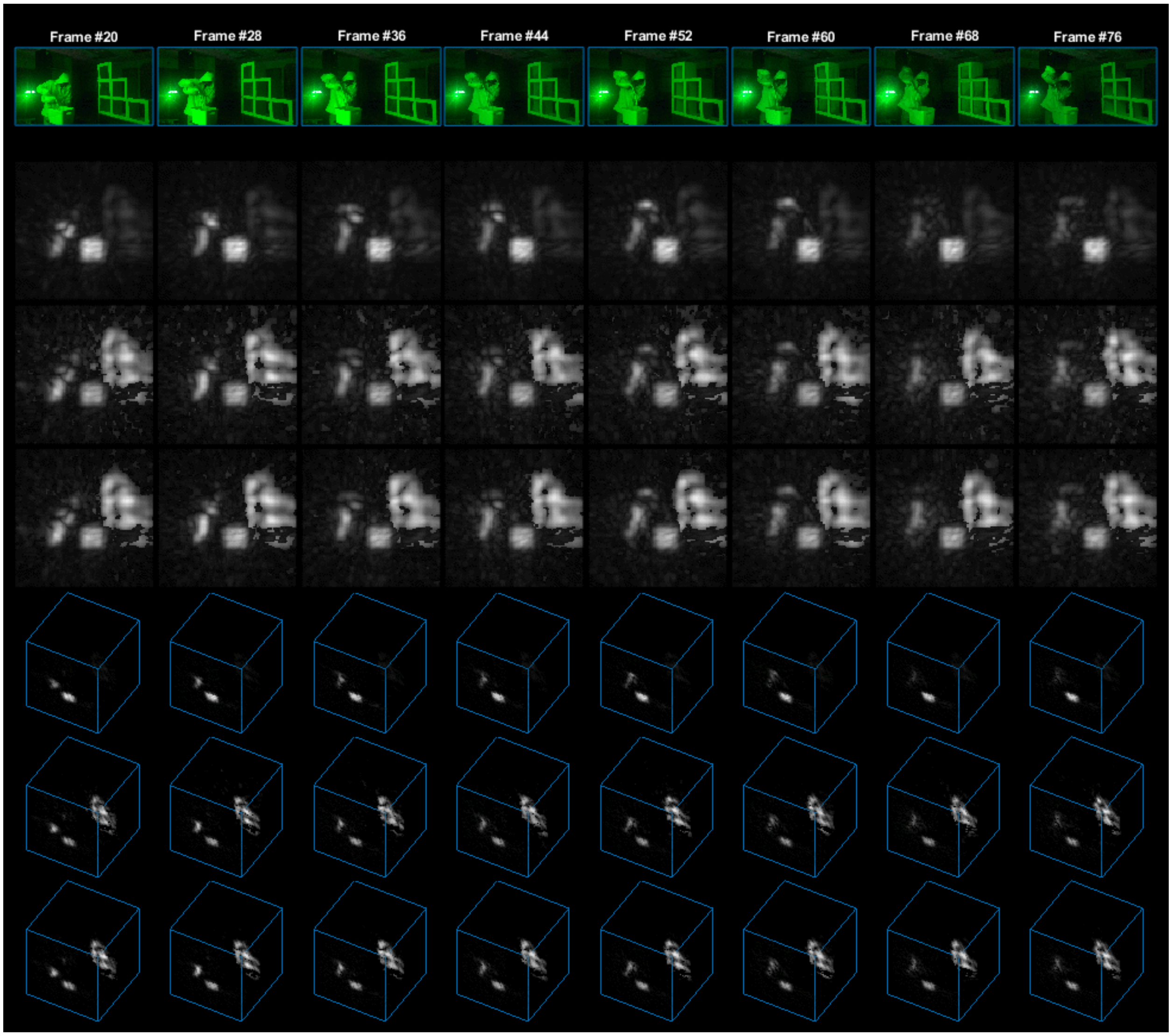

Supplementary Figure 12: **Video: "Milk".** Ground truth, RSD, RSD with intensity correction, RSD with intensity correction and averaging.

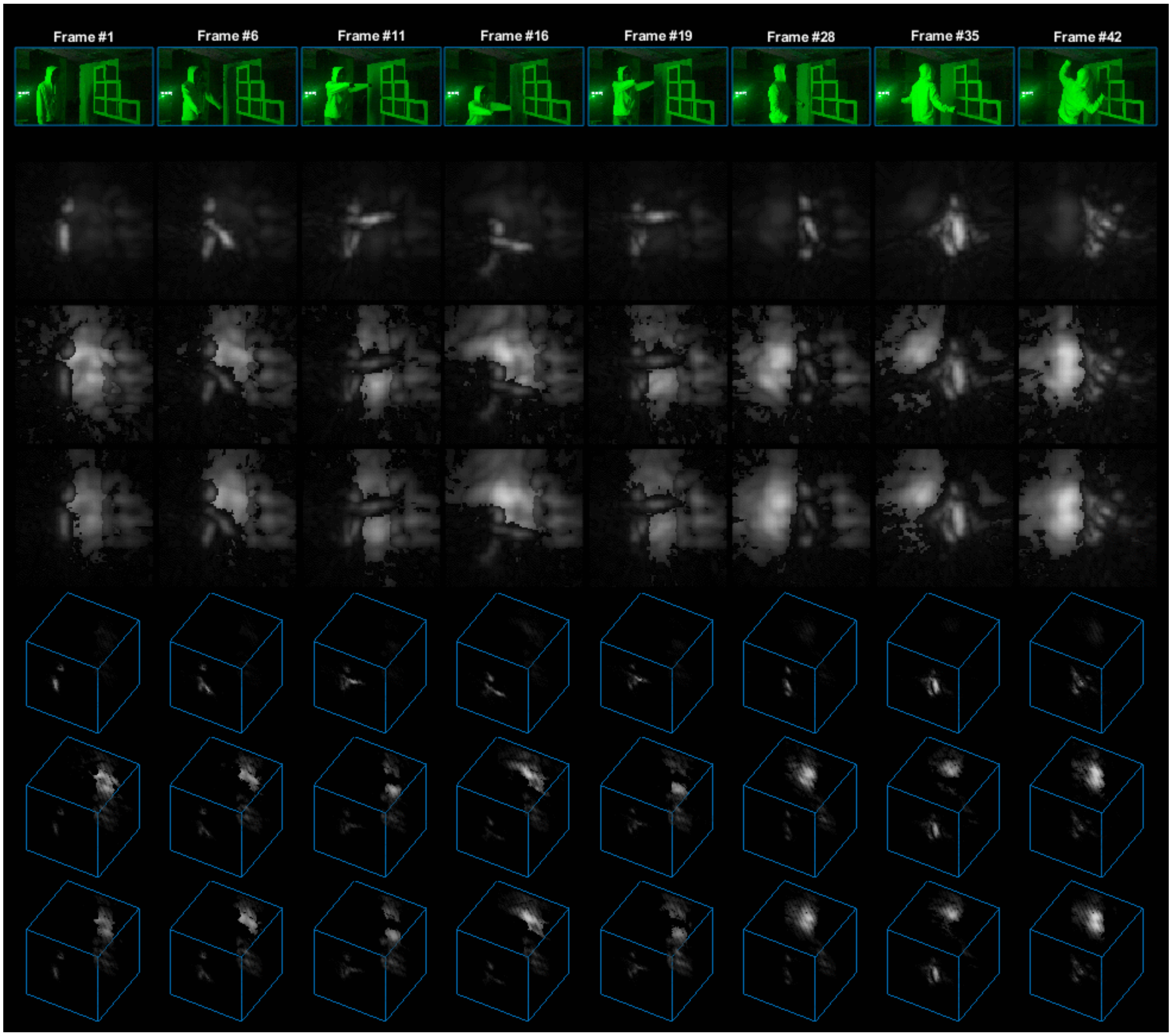

Supplementary Figure 13: **Video: "Exercise".** Ground truth, RSD, RSD with intensity correction, RSD with intensity correction and averaging.

6 Supplementary References

1. Martin H. Weik. *Lambert's cosine law*, pages 868–868. Springer US, Boston, MA, 2001.

2. Matthew O'Toole, David B Lindell, and Gordon Wetzstein. Confocal non-line-of-sight imaging based on the light-cone transform. *Nature*, 555(7696):338, 2018.

3. Genevieve Gariepy, Francesco Tonolini, Robert Henderson, Jonathan Leach, and Daniele Faccio. Detection and tracking of moving objects hidden from view. *Nature Photonics*, 10(1):23, 2016.

4. Xiaochun Liu, Ibón Guillén, Marco La Manna, Ji Hyun Nam, Syed Azer Reza, Toan Huu Le, Adrian Jarabo, Diego Gutierrez, and Andreas Velten. Non-line-of-sight imaging using phasor-field virtual wave optics. *Nature*, 572(7771):620–623, 2019.

5. Xiaochun Liu, Sebastian Bauer, and Andreas Velten. Phasor field diffraction based reconstruction for fast non-line-of-sight imaging systems. *Nature Communications*, 11(1):1645, April 2020.
