# Supplementary material for: Low-latency time-of-flight non-line-of-sight imaging at 5 frames per second
Source: Nat Commun. 2021 Nov 11;12:6526. doi: 10.1038/s41467-021-26721-x (PMC8586255; doi:10.1038/s41467-021-26721-x)
Supplement: Supplementary file 3 — Description of Additional Supplementary Files [file 41467_2021_26721_MOESM3_ESM.docx]

**Description of Additional Supplementary Files:**

**Supplementary Video:**

It is a demonstration of the system and its live (low-latency) performance
